# Identification of disease-associated loci using machine learning for genotype and network data integration

**DOI:** 10.1093/bioinformatics/btz310

**Published:** 2019-05-09

**Authors:** Luis G Leal, Alessia David, Marjo-Riita Jarvelin, Sylvain Sebert, Minna Männikkö, Ville Karhunen, Eleanor Seaby, Clive Hoggart, Michael J E Sternberg

**Affiliations:** 1 Department of Life Sciences, Centre for Integrative Systems Biology and Bioinformatics, Imperial College London, London SW7 2AZ, UK; 2 Center for Life Course Health Research, Faculty of Medicine, University of Oulu, Oulu FI-90014, Finland; 3 Biocenter Oulu, University of Oulu, Oulu 90220, Finland; 4 Unit of Primary Health Care, Oulu University Hospital, Oulu 90220, Finland; 5 Department of Epidemiology and Biostatistics, MRC-PHE Centre for Environment and Health, School of Public Health, Imperial College London, London W2 1PG, UK; 6 Department of Life Sciences, College of Health and Life Sciences, Brunel University London, Middlesex UB8 3PH, UK; 7 Program in Medical and Population Genetics, Broad Institute of MIT and Harvard, Cambridge, MA, USA; 8 Department of Medicine, Imperial College London, London W2 1PG, UK

## Abstract

**Motivation:**

Integration of different omics data could markedly help to identify biological signatures, understand the missing heritability of complex diseases and ultimately achieve personalized medicine. Standard regression models used in Genome-Wide Association Studies (GWAS) identify loci with a strong effect size, whereas GWAS meta-analyses are often needed to capture weak loci contributing to the missing heritability. Development of novel machine learning algorithms for merging genotype data with other omics data is highly needed as it could enhance the prioritization of weak loci.

**Results:**

We developed cNMTF (corrected non-negative matrix tri-factorization), an integrative algorithm based on clustering techniques of biological data. This method assesses the inter-relatedness between genotypes, phenotypes, the damaging effect of the variants and gene networks in order to identify loci-trait associations. cNMTF was used to prioritize genes associated with lipid traits in two population cohorts. We replicated 129 genes reported in GWAS world-wide and provided evidence that supports 85% of our findings (226 out of 265 genes), including recent associations in literature (*NLGN1*), regulators of lipid metabolism (*DAB1*) and pleiotropic genes for lipid traits (*CARM1*). Moreover, cNMTF performed efficiently against strong population structures by accounting for the individuals’ ancestry. As the method is flexible in the incorporation of diverse omics data sources, it can be easily adapted to the user’s research needs.

**Availability and implementation:**

An *R* package (*cnmtf*) is available at https://lgl15.github.io/cnmtf_web/index.html.

**Supplementary information:**

Supplementary data are available at *Bioinformatics* online.

## 1 Introduction

Polygenic diseases result from the contribution of multiple loci but only those with large effect size are detected by traditional methods for Genome-Wide Association Studies (GWAS) (Arkin *et al.*, 2014; Bush and Moore, 2012). Although regression models (RMs) have been useful for discovering significant single nucleotide variant (SNV) associations, most of these variants explain just a fraction of the phenotypic variability and several loci with a small effect size on the trait are not being captured (Auer and Lettre, 2015; Zuk *et al.*, 2012). It is also evident that genotyping data alone cannot expand our understanding on how SNVs alter protein sequences, mRNA transcript stability, transcription factors and ultimately, the observed phenotypes. Instead, data integration frameworks must be developed to analyze other sources of omics data and for pinpointing variants that associate additively with the trait.

Here, we address the problem that causal loci are underpowered in single-SNV analysis because few subjects carry the same causal variants. Thus, for most of the subjects, different causal variants are observed within the gene region and in genes participating in related cellular functions (Leiserson *et al.*, 2013). This problem is of particular interest in the area of network-assisted analysis of GWAS data (Jia and Zhao, 2014), where the prioritization of candidate genes is enhanced by aggregating GWAS summary statistics onto protein–protein interactions (PPI) or metabolic pathways, followed by searching connected modules within the network (Lee *et al.*, 2011; Leiserson *et al.*, 2013; Liu *et al.*, 2017a, b; Rossin *et al.*, 2011). Despite of their applications, these methods ignore the presence of confounding variables in the input data (e.g. subjects’ genetic background), and the information lost by aggregating SNV-association signals into the gene level.

Taking into account these limitations and the need of integrative frameworks for GWAS and omics data, we developed cNMTF (corrected non-negative matrix tri-factorization). Based on the principle that causal genes participate in similar cellular functions, cNMTF uses the PPI network (PPIN) to share genotyping information among SNVs and identifies novel genes closer to known loci. The method relies on machine learning techniques for clustering, which have been used for studying high-dimensional heterogeneous data in systems biology (Gligorijević and Pržulj, 2015; Gligorijević *et al.*, 2014, 2016a, b; Hwang *et al.*, 2012; Xiao *et al.*, 2018; Žitnik and Zupan, 2015). Our algorithm is itself an extension of non-negative matrix factorization (NMF) into the context of GWAS, allowing the clustering of subjects with common phenotypes, and simultaneously, the prioritization of data entries (e.g. genes, SNVs) acting jointly on the phenotype.

A first major feature of cNMTF resides in the integration of heterogeneous sources of information: genotypes, phenotypes, subjects’ ancestry, the predicted deleteriousness effect of SNVs and PPIs. The integration is performed using raw genotyping data rather than aggregated GWAS statistics, so there is no loss of information when moving into the gene level. A second novelty of cNMTF is its ability for correcting the results against population structures via kernel functions. To our knowledge, cNMTF is the first framework that brings together matrix factorization and kernel methods to solve the problem of confounding factors in heterogeneous data analysis, and adheres to similar improvements for support vector machines (SVMs) (Li *et al.*, 2011).

Our algorithm was tested on three lipid traits: low-density lipoproteins cholesterol (LDL-C), high-density lipoproteins cholesterol (HDL-C) and triglycerides (TG) levels. A number of studies have placed these traits as strong determinants of cardiovascular disease risk; however, the genetic architecture of lipid metabolism is far from understood (Paththinige *et al.*, 2017). This offers an opportunity to identify new variants and loci using cNMTF.

We obtained genotyping data from two consortia to evaluate our method: The Northern Finland Birth Cohort 1966 (NFBC1966), a relatively genetically homogeneous cohort of Finnish individuals with lipid traits associations reported by Sabatti *et al.* (2009); and a cohort of white American subjects of diverse European ancestry obtained from the Electronic Medical Records and Genomics network (eMERGE) (McCarty *et al.*, 2011).

Across cohorts, cNMTF significantly prioritized a mean of 93 variants and 44 genes per trait (*P_cNMTF_* < 0.005), replicating a total of 23 variants from prior studies. We showed that cNMTF complements results of RMs by capturing patterns of association that do not reach statistical significance in the single-SNV methods. In addition, we proved the capabilities of our algorithm for identifying population structures in the data and correcting the results against false positive associations.

## 2 Materials and methods

### 2.1 Input matrices

**Fig. 1.**
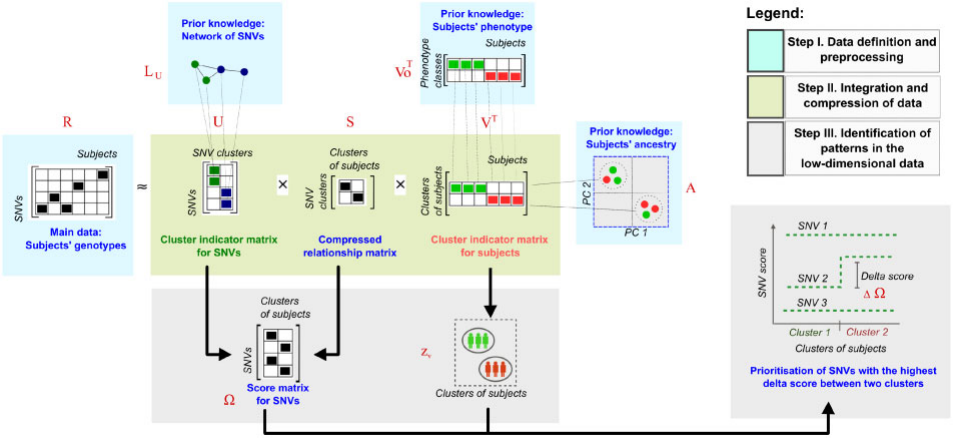
Main steps in cNMTF. Step 1: The algorithm takes four sources of data in the input: ***R*** is a relationship matrix for the genotyping data. $L_{U}$ is the Laplacian matrix of an SNV–SNV network. $V_{o}$ is a phenotype matrix. ***A*** is a kernel similarity matrix encoding the population origin of the subjects. Step 2: The ***R*** matrix is approximated as the product of low-dimensional matrices ***U***, ***V*** and ***S***. Here, we use $L_{U},\;V_{o}$ and ***A*** to penalize the factorization and guide the solutions of ***U*** and ***V***. Step 3: The dimensional reduction provides information for clustering tasks, so ***U*** and ***V*** are taken as cluster indicator matrices for SNVs and subjects, respectively. Simultaneously, we compute the product of ***U*** and ***S*** to generate a score matrix Ω. This matrix summarizes the effect of single SNVs on clusters of subjects with specific phenotypes and can be used to prioritize the SNVs. When SNV scores are compared between clusters we observe the relative importance of each SNV on a trait; therefore, those SNVs with high delta score, ΔΩ, can be prioritized for further analysis

cNMTF is a data integration framework that scores SNV-trait associations and finds clustering patterns in the genotypes of subjects. Its search is guided with prior knowledge on the phenotypes, subjects’ ancestry and molecular SNV data (Fig. 1).

The algorithm takes a matrix of genotypes, $R_{\left(n\times m\right)}$, that encodes the number of recessive allele copies carried by the ***m*** subjects across ***n*** SNVs. Considering $G_{i,j}$, the genotype of patient *j* in the SNV *i* with alleles *A* and *B*; then, $R_{i,j}=0$ if $G_{i,j}=\mathrm{AA},\;R_{i,j}=1$ if $G_{i,j}=\mathrm{AB}$ and $R_{i,j}=2$ if $G_{i,j}=\mathrm{BB}$. Thus, we set $R_{\left(n\times m\right)}$ to contain the genotypes under an additive genetic model (Lewis and Knight, 2012).

We decompose the relationship matrix ***R*** in three low-dimensional matrices $U_{\left(n\times k_{1}\right)},\;V_{\left(m\times k_{2}\right)}$ and $S_{\left(k_{1}\times k_{2}\right)}$:

(1) $$R≅\mathrm{US}V^{T}$$

This dimensional reduction is provided by rank parameters *k*_1_ and *k*_2_$\left(k_{1}\ll n,k_{2}\ll m\right)$, which are chosen *a priori*. Both $U_{\left(n\times k_{1}\right)}$ and $V_{\left(n\times m\right)}$ can be seen as cluster indicator matrices for SNVs and subjects, respectively, where *k*_1_ and *k*_2_ are the number of clusters. For example, each subject is assigned to a cluster by finding the maximum entries in the rows of ***V*** (i.e. the values in each row of ***V*** represent the importance or ‘learned weights’ of a given subject with respect to a subject cluster). On the other hand, $S_{\left(k_{1}\times k_{2}\right)}$ is a compressed version of ***R*** that describes the importance of a given SNV cluster with respect to a subject cluster (Gligorijević and Pržulj, 2015).

One of the novelties of cNMTF resides in the definition of three sources of information to guide the solution of ***U***, ***V*** and ***S*** as follows:

#### 2.1.1 Subjects’ phenotypes ($V_{o}$)

The solution of ***V*** is guided with prior knowledge about subjects’ clinical phenotypes. That information is added through a phenotype matrix $V_{o\left(m\;\times \;k_{2}\right)}$, of *m* subjects by *k*_2_ phenotype categories. We set $k_{2}=2$ because we are studying binary phenotypes or cases–control designs. The entries of this matrix are $V_{o[j,1]}=1$ if patient *j* is a control ($V_{o[j,1]}=0$ otherwise) and $V_{o[j,2]}=1$ if patient *j* is a case ($V_{o[j,2]}=0$ otherwise).

#### 2.1.2 SNV–SNV network ($L_{U}$)

The solution of ***U*** is guided with prior knowledge about the SNV potential to disrupt the function of interacting proteins. Our hypothesis is that SNVs do not act independently but their effects are dependent on other polymorphisms elsewhere in the genome. Therefore, we constructed an SNV–SNV network containing two kinds of nodes: *damaging* variants and *candidate* variants (Supplementary Fig. S1). The set of *damaging* variants correspond to SNVs with high or moderate impact in the genome according to ENSEMBL (e.g. frameshift variant, stop gained variants). In addition, SNVs annotated as deleterious by Sift or Polyphen (VEP-ENSEMBL query, McLaren *et al.*, 2016) and SNVs known to be associated with the trait in the GWAS catalogue were labelled as *damaging* variants. All the remaining SNVs were labelled as *candidate* variants. Then, we connected two SNVs in the network if both SNVs are harboured by the same gene. *Damaging* SNVs from different genes were also connected because they are more likely to disrupt the PPI (BioGrid multi-validated interaction list). The number of candidate and damaging variants is reported in Table 1.

**Table 1.** Results of cNMTF applied on serum lipid levels

Procedure	Variable	Finnish			White American		
		LDL-C	HDL-C	TG	LDL-C	HDL-C	TG
Pre-processing phenotype data	Cut-off level for controls (mg/dl)	<100	>60	<150	<100	>60	<150
	Cut-off level for cases (mg/dl)	≥160	<40	≥200	≥160	<40	≥200
	Number of subjects in the input:	1711	1920	3780	446	605	1300
	Number of controls	1344	1775	3635	308	202	1214
	Number of cases	367	145	145	138	403	86
Pre-processing genetic data	Number of SNVs in the input:	6945	7158	7620	9888	12 476	8662
	Candidate variants	6703	6910	7407	9626	12 179	8446
	Damaging variants	242	248	213	262	297	216
	Number of genes in the input:	510	724	389	536	773	441
	Seed genes^a^	136	180	123	142	193	139
	Candidate genes in the PPIN	374	544	266	394	580	302
Results	Number of SNVs prioritized:	87	80	93	110	117	71
	Number of genes prioritized:	40	41	25	54	65	40
	Prioritized candidate genes	21	14	6	36	33	26
Top candidate gene prioritized^b^	Gene name	*PDS5B*	*IKZF5*	*DOCK8*	*BIRC2*	*LGR4*	*MLLT1*
	SNV	*rs590383*	*rs13353058*	*rs10968671*	*rs12275349*	*rs7936621*	*rs8099971*
	Alleles (*A, B*)	C, T	A, G	G, T	G, A	G, A	T, C
	*P_cNMTF_*	$3\times 10^{-4}$	$1\times 10^{-3}$	$7\times 10^{-6}$	$3\times 10^{-4}$	$2\times 10^{-4}$	$1\times 10^{-4}$
	ΔΩ	3.7	−3.8	−5.3	4.4	4.0	4.0

a Refers to associations reported in GWAS catalogue under the genome-wide significance threshold $P<1\times 10^{8}$.

b This section shows the most significant novel gene not reported in GWAS catalogue. It lists the SNVs with the lowest *P*-value within each gen (*P_cNMTF_*) and their delta score (ΔΩ). The complete list of prioritized genes and SNVs is annexed in Supplementary Files S2 and S3.

Afterwards, the network was weighted to give preference to edges between *damaging* variants. The weighting aims to reduce bias in the node degree when genes harbour thousands of variants, so the edges were divided by N−1, where *N* is the number of variants in the gene.

#### 2.1.3 Subjects’ ancestry (*A*)

The solution of ***V*** is corrected for the individual’s ancestry or population origin. We used the Hilbert–Schmidt independence criterion (HSIC), which has been previously used for correcting SVMs (Li *et al.*, 2011).

The HSIC is a measure of statistical independence between two random variables (*X* and *Y*) computed in terms of kernel functions (Li *et al.*, 2011). Let *X* be a random variable from the domain X, with feature space F and associated kernel k:X×X→R. Let *Y* be a random variable from the domain Y, with feature space G and associated kernel a:Y×Y→R. The empirical estimator of HSIC for a finite sample of points *x* and *y* is shown to be:

(2) HSIC(X,Y)∝tr(KHAH)

where ***K*** and ***A*** are the kernel matrices on the random variables *X* and *Y*, given by: $K_{i,j}=k(x_{i},x_{j})$ and $A_{i,j}=a(y_{i},y_{j})$. ***H*** is a centring matrix: $H=\delta_{i,j}-\frac{1}{m}$, where $\delta_{i,j}=1$ if *i *=* j* and $\delta_{i,j}=0$ otherwise. The size of these matrices is *m *×* m*.

Because small values of HSIC are expected when *X* and *Y* are independent, we define the following kernel matrices:

- Kernel matrix $K_{i,j}$: Suppose *m* subjects with clustering information vectors ($v_{1},\ldots ,v_{m}$). The $K_{i,j}=k(v_{i},v_{j})$ is a kernel matrix generated by the linear kernel *k* on the clustering information: $K=VV^{T}$
- Kernel matrix $A_{i,j}$: Generated by the kernel *a* on the population structure information. The population origin is frequently unknown, so it must be inferred via principal components analysis using the similarity between subjects in the principal components space.

### 2.2 The objective function

To obtain ***U***, ***S*** and ***V***, we solve an optimization problem denoted by the objective function, *J_cNMTF_*:

(3) $$\begin{array}{c} \underset{U\geq 0,S\geq 0,V\geq 0}{\min}J_{\mathrm{cNMTF}}=||R-\mathrm{US}V^{T}|{|}_{F}^{2}\\ +\gamma_{1}\cdot \mathrm{tr}(U^{T}L_{U}U)\\ +\gamma_{2}\cdot ||\mathrm{V-}V_{o}|{|}_{F}^{2}\\ +\gamma_{3}\cdot \mathrm{tr}(VV^{T}\mathrm{HAH}) \end{array}$$

where $||\cdot |{|}_{F}^{2}$ refers to the Frobenius norm, tr(·) is the trace of a matrix and $\cdot^{T}$ is the transpose of a matrix. The goal in Equation (3) is to minimize the difference between the genotyping data (***R***) and the new matrices using the Frobenius norm, while guiding the solution with the penalization terms:

- The phenotype matrix ***V*_*o*_** maximizes the separation between subjects with different phenotypes in the term $||V-V_{o}|{|}_{F}^{2}$, while *γ*_2_ weights the influence of the phenotype.
- The SNV–SNV network is integrated in the form of a graph Laplacian, $L_{U\left(n\;\times \;n\right)}=D_{U\left(n\;\times \;n\right)}-W_{U\left(n\;\times \;n\right)}$, where $W_{U}$ is the weighted adjacency matrix and $D_{U}$ is the diagonal degree matrix of $W_{U}$. Hence, a new term, $\mathrm{tr}(U^{T}L_{U}U)$, guides the solution of ***U*** in the objective function and *γ*_1_ weights the influence of the network. We also tested the algorithm when using a normalized graph Laplacian: $L_{U}=I-D_{U}{}^{-\frac{1}{2}}W_{U}D_{U}{}^{-\frac{1}{2}}$, where $I_{\left(n\times n\right)}$ is the identity matrix with ones on the main diagonal.
- The HSIC term $\mathrm{tr}(VV^{T}\mathrm{HAH})$ is added to correct for population structures and parameter *γ*_3_ weights that regularization.

In Supplementary File S1, Sections S4–S6, the optimization problem is solved using iterative update rules (Lee and Seung, 2000). We show how to achieve unique solutions and robust prioritizations for the different traits by running the algorithm multiple times with different initializations, extracting the clustering solutions from each repetition and combining these results to conform a consensus solution. The computational complexity of this minimization problem is shown to be quadratic in the number of SNVs, $O(n^{2})$.

### 2.3 Delta scores, ΔΩ

The minimization of the objective function (Equation (3)) allows us to find the clusters of subjects from ***V***. Simultaneously, we use ***U*** and ***S*** to prioritize SNV-trait associations. We summarize the number of copies of recessive alleles and the connectivity patterns of the network into an SNV score, $\Omega_{s,l}$, which tells how important is the variant *s* for a given cluster of subjects *l* (Equation (4)).

(4) $$\Omega_{\left(n\times k_{2}\right)}=\mathrm{US}$$

After comparing the SNV scores between clusters enriched in controls and cases (Equation (5)), we can prioritize variants

(5) $$\Delta \Omega_{s}=\Omega_{s,\mathrm{controls}}-\Omega_{s,\mathrm{cases}}$$

The delta score captures the overall effect of the SNV in the cluster of controls. For instance, a positive $\Delta \Omega_{s}$ means a *protective* effect of the recessive allele *B*, because the controls are carrying more copies of the recessive allele when compared with the cases. In contrast, negative $\Delta \Omega_{s}$ mean that recessive alleles are *detrimental* and preferentially observed in cases.

### 2.4 Significance of the Delta scores, ΔΩ

We assessed the significance of the observed ΔΩ in order to compare objectively our results [i.e. a *P*-value for the SNV-trait association is provided (*P_cNMTF_*)]. For each trait, the null distribution of ΔΩ was generated by running cNMTF with 1 000 randomizations of phenotypes. We set a significance level, α=0.01, to prioritize approximately 100 variants in the tails of the distribution. Thus, cut-off points at $\left(\frac{\alpha }{2},1-\frac{\alpha }{2}\right)$ are obtained from the cumulative distribution function (Supplementary Fig. S3). These cut-off points are estimated for every genetic dataset, trait and setting of the algorithm.

### 2.5 Parameters selection

There are two sets of parameters to be chosen sequentially. In a first step, we explore the structure of the data to select the number of clusters of SNVs and subjects, *k*_1_ and *k*_2_, respectively. We selected $k_{2}=2$ because we follow a case–control study design. On the other hand, the selection of *k*_1_ is based on a grid search while tracking the cluster stability. We used a dispersion coefficient to summarize the consistency of clustering assignments throughout repetitions of the algorithm (Kim and Park, 2007) (Supplementary Fig. S5.A).

The second step is to explore the contribution of data sources (***R***, $L_{u}$, ***A*** and $V_{o}$), while changing the thresholding parameters. Here, the optimal *γ*_1_ maximizes the number of known loci-trait associations in the results, whereas optimal *γ*_2_ and *γ*_3_ maximize the separation of case–controls. See Supplementary File S1, Sections S8 and S9 and Figure S4 for details on the grid search and how we quantified the transference of information.

### 2.6 Genotyping data and phenotypes

Array genotyping data and clinical variables from 5 402 Finnish and 6 100 white American subjects (NFBC1966 and eMERGE cohorts) were pre-processed as described in Supplementary File S1 and (Section S1). We also simulated genotyping data with embedded population structures.

With regards to the phenotypes, we focussed on three lipid traits: LDL-C, HDL-C and TG. For each individual we obtained the following clinical variables which are potential confounders of the associations: age, alcohol consumption, smoking status, body mass index (BMI), BMI at birth, pregnancy and use of oral contraceptives (Supplementary Table S1).

As our method is aimed at the study of categorical phenotypes, we categorized the trait by using cut-off levels and compared subjects at extreme categories. For LDL-C, we divided our cohort in controls (subjects with LDL-C < 100 mg/dl) and cases (subjects with LDL-C > 160 mg/dl). These cutoffs were chosen following the optimal and high LDL-C levels for cardiovascular risk prevention (Supplementary Table S2, ATP III guidelines). Similarly, for the other traits, subjects were divided in cases and controls using the cutoffs reported in Table 1. Subjects with intermediate lipid levels were excluded from our analysis.

We subtracted the effect of confounders from the phenotype by fitting a multiple RM. The residuals of the model were used as a corrected phenotype that is explained by the genetic component of the subjects rather than by the confounders (Supplementary File S1 and Section S3). Notably, the confounding effect from subjects’ ancestry is corrected simultaneously within the cNMTF algorithm, constituting one of the main contributions of this work.

### 2.7 A subset of SNVs in the relationship matrix (*R*)

**Fig. 2.**
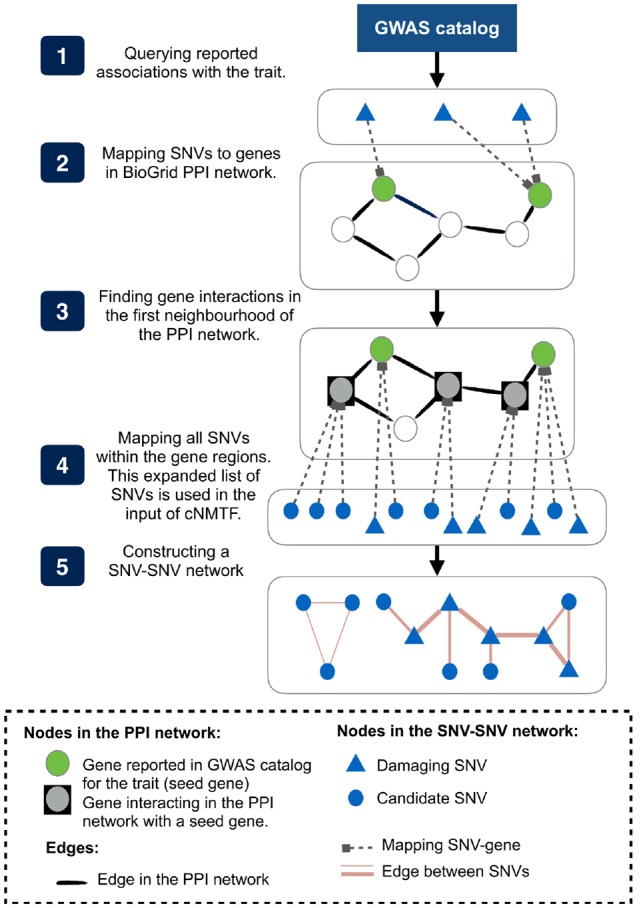
Defining a subset of SNVs to analyze with cNMTF. (**1**) Reported SNV-trait associations are queried from GWAS catalogue and (**2**) mapped to genes in a PPIN. (**3**) The list of genes is expanded using their interactions in the first neighbourhood of the PPIN. (**4**) All variants located in the expanded list of genes were selected to conform the subset of SNVs, and later included in the SNV–SNV network

We aimed at reducing the universe of variants to a meaningful subset that could explain the phenotype. This pre-selection of SNVs reduces the data noise, increases the performance of the method and alleviates the computational complexity during the matrix operations (Fig. 2).

First, we queried the GWAS catalogue for all known variants associated with the trait (under the genome-wide significance threshold $P<10^{-8}$) (MacArthur *et al.*, 2017) (available at: www.ebi.ac.uk/gwas, accessed 1 August 2018, version 1.0.2). These variants can be seen as a seed group that will be expanded to a larger group of variants. To achieve this, we identified the genes harbouring known associations (seed genes) and mapped them onto a PPIN (BioGrid interactions validated by multiple publication sources and experimental systems; available at: https://thebiogrid.org, accessed 1 August 2018, version 3.4.162). Please note that seed genes with no interactions in the PPIN are analyzed as well (i.e. isolated nodes).

Then, variants located in the region of seed genes and variants located in the interacting gene partners (first neighbourhood in the PPIN) were selected to form the subset of SNVs. The *gene region* is determined by the start and stop genomic coordinates of the gene. Hence, the final genotyping data input, ***R***, contained on average 8 800 SNVs per trait-cohort analyzed.

We further compared the results of the algorithm when clumping SNVs in high linkage disequilibrium (LD) from the input. The SNV clumping is a pre-processing step where a proxy SNV is chosen to represent a gene region in high LD ($r^{2}>0.5$) whereas non-proxy SNVs are excluded from that gene region. The proxy variant is not selected at random but we select the variant most associated with the trait using Logistic Regression Models (LRM) (Euesden *et al.*, 2018). For purposes of the SNV–SNV network construction, non-proxy SNVs are removed but their functional impact (i.e. *damaging* variant) is inherited to the proxy SNV in the network, so this information is not lost during the clumping. On average, this pre-processing step reduces the data input to ∼7 800 SNVs.

## 3 Results

The dispersion of delta scores for LDL-C is displayed in Supplementary Figure S7, where the prioritized variants are those above and below the significance cut-off points. The delta score indicates the trend of the association: (+) *protective* effect of the recessive allele *B* towards optimal conditions of LDL-C; (−) *detrimental* effect of the recessive allele *B* leading to abnormal conditions of high LDL-C.

We mapped the prioritized variants to genes and ranked the genes using the variant with lowest *P_cNMTF_*. A mean of 93 variants and 44 genes was prioritized per cohort and trait (Table 1).

### 3.1 Prioritization of SNVs

We replicated a total of 23 genome-wide significant associations from GWAS catalogue ($P<10^{-8}$) using smaller sample sizes than the original studies. For LDL-C, we replicated two out of four significant associations reported by Sabatti *et al.* (2009) in the Finnish cohort: *rs174546*, *rs1535*; and two more associations reported in other studies (*rs2228671*, *rs10490626*) (Aulchenko *et al.*, 2009; Teslovich *et al.*, 2010). For HDL-C and TG, the numbers were broadly similar; our method recalled 7 and 12 known associations in both cohorts, respectively (Supplementary Figs S8–S11).

We compiled the information for prioritized SNVs, including their delta score, *P_cNMTF_*, minor alleles, genes and consequences on the transcript (Supplementary File S2). It was found that 19 out 23 variants conserve the trend reported in the original GWAS. This result supports the codification of recessive alleles in the input matrix ***R***, and the mathematical formulation of the scores from low-dimensional matrices, because there is an agreement between the sign of the delta score and the sign of beta coefficients from RMs.

In the formulation of the SNV–SNV network, we labelled the variants as damaging or candidates. The set of prioritized variants contains 7% damaging variants, compared to 3% in the input network. Thus, an enrichment of damaging variants is observed in the results ($P=8\times 10^{-8}$), but still a large proportion of prioritized variants have no evidence of impact in the protein (e.g. synonymous variants). In fact, most of the SNVs are located in intronic regions or affect non-coding transcripts with no further consequences in the protein (Supplementary Fig. S12). Among the SNVs with high impact are *rs2228671*, a stop gain variant in the LDL receptor (*LDLR*), and 18 missense mutations (Supplementary File S2).

The LD structure among variants was considered in the pre-processing steps and we compared the lists of prioritized genes with/without SNV clumping. We found that results are highly robust in the trait-cohort analyses, where ∼90% of prioritized genes were preserved between both settings (Supplementary Fig. S17 and File S6). This can be explained by the clustering of SNVs taking place in matrix ***U*** (Equation (1)). Therefore, SNVs in LD are clustered and weighted together because their profiles are analogous in cases/controls and they also reside in the same gene region (they are connected in the SNV–SNV network). For example, *rs*174556 and *rs*1535 are in LD ($r^{2}=0.92$) in gene *FADS1*. Their prioritization scores (without clumping) are 4.004 and 3.444, respectively, for association with LDL-C in Finnish individuals. If clumping is performed, *rs*1535 is filtered out from the input. The proxy SNV is *rs*174556, whose score slightly changes from 4.004 to 4.006 and *FADS1* is still prioritized ($P_{\mathrm{cNMTF}}=3\times 10^{-4}$). In general, the method achieves similar performance with either a set of high LD SNVs in the input or just proxy SNVs representing the same loci.

### 3.2 Prioritized genes

We consider that cNMTF is a complementary tool to explore hidden patterns of association that usually pass undetected with LRM. To make this a fair comparison between methods, we choose the same level of significance for SNV prioritization (α=0.01), and mapped the SNVs to genes. Then, we compared the performance in retrieving significant genes from the Global Lipids Genetics Consortium (GLGC, European-ancestry individuals) (Supplementary Fig. S14) (Willer *et al.*, 2013). For cNMTF, 99 out of 265 prioritized genes are significant in GLGC (Precision, PR = 37%); whereas for LRM, 136 out of 394 genes are significant (PR = 35%).

**Fig. 3.**
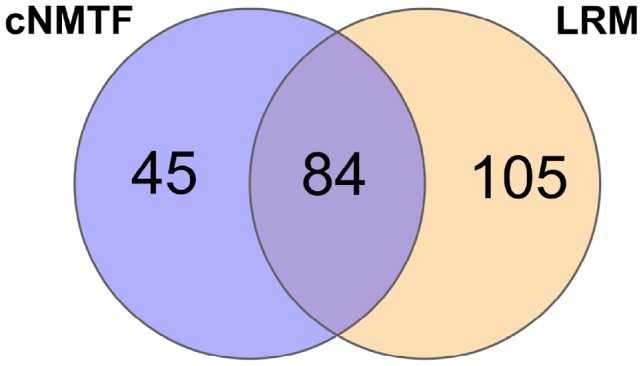
Enhancing gene discovery with cNMTF. Prioritized genes across trait-cohorts are totalized and intersected with the results of LRM. Only genes benchmarked against GWAS catalogue are counted. In Supplementary Figure S15, we present Venn diagrams for specific trait-cohorts

In a second step, the results were benchmarked against the lipid-associated genes in GWAS catalogue, which includes curated information from GLGC and all published GWAS world-wide (Supplementary Fig. S15). The PR increased to 49% and 48% for cNMTF and LRM, respectively. Among the true positives, there are 45 genes captured exclusively by cNMTF, 105 genes captured only by LRM and 84 genes in the intersection of both methods (Fig. 3). This supports our conclusion that cNMTF and LRM results are complementary and mutually enhance gene discovery for GWAS.

As seen in the dispersion plots of Supplementary Figure S14, one of the strengths of cNMTF is on leveraging genes with moderate association through data integration. We compared the *P*-values between both methods and found that 29% of the genes prioritized by cNMTF nearly reach the significance level in LRM ($0.01<P_{\mathrm{LRM}}<0.10$). These genes would have been disregarded due to insufficient statistical power in the case–control samples. cNMTF, on its own, makes use of the PPIN to share association signals between variants, which is the strength of this machine learning method over single-SNV analyses.

**Fig. 4.**
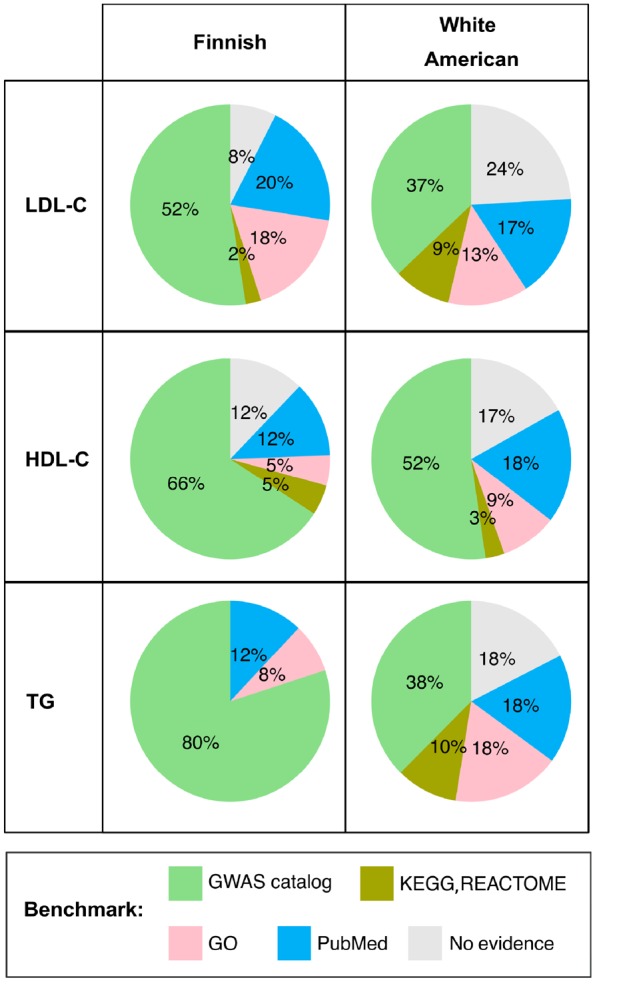
Benchmarking prioritized genes by cNMTF. Percentage of genes with known functional implications in the lipid metabolism. The search for evidence includes GWAS catalogue (the strongest benchmark for associations), KEGG, Reactome, GO and finalizes in PubMed

We searched for additional evidence to support our findings with different benchmarks (Fig. 4):

1. The benckmarking of cNMTF with GWAS catalogue gave us evidence for 129 out of 265 genes (49%). We noted that despite the low proportion of seed genes in the input, a significant enrichment of them is obtained in most of the outputs (Supplementary Fig. S13). This means that our results are not biased by the subset of variants in the input, and that cNMTF is truly differentiating signals of association from any noise generated by the PPIN.
2. Further exploration of GWAS catalogue was conducted to include closely related traits (e.g. ‘hypertriglyceridemia’, ‘total cholesterol levels’. The complete list of 101 related traits is annexed in Supplementary File S5). This procedure gave us evidence of association for eight more genes (3%). For instance, the gene *CARM1* is reported with the combined phenotype ‘C-reactive protein levels/LDL-C levels (pleiotropy)’ (*rs1529711*, $P<10^{-8}$) (Ligthart *et al.*, 2016). Also, CARM1 association with LDL-C was updated recently (*rs2304088*, $P<10^{-23}$, GWAS catalogue, February 2019, retrospective validation).
3. In the remaining genes not reported in GWAS catalogue, we searched for functional implications in GO, KEGG, Reactome and OMIM database. We found 45 genes (17%) related to biological processes/pathways in the lipid metabolism (e.g. fatty acid biosynthesis). Some of them were significantly enriched in novel genes (*P *< 0.05, Supplementary Table S5). No additional evidence was observed in OMIM for lipid-related traits because most of the OMIM entries overlap with GWAS catalogue.
4. A literature search was conducted in PubMed to find regulation and epigenetics evidence that could connect the novel genes with altered lipid metabolism. We observed evidence in 44 genes (17%), including regulators of important genes [*DAB1-LDLR*; *SNTB2-ABCA1* (Bock *et al.*, 2003; Hebel *et al.*, 2015)], and studies in other organisms [*DLGAP1* in mice, *NLGN1* in ducks (Chadwick et al., 2010; Zhu *et al.*, 2019)]. Noteworthy, the association of *NLGN1* with total cholesterol was reported by this year. A short description of our findings is presented in Supplementary File S3 for each gene.

All in all, we collected evidence that supports 226 genes (85%).

### 3.3 Patterns of interaction in the PPIN

**Fig. 5.**
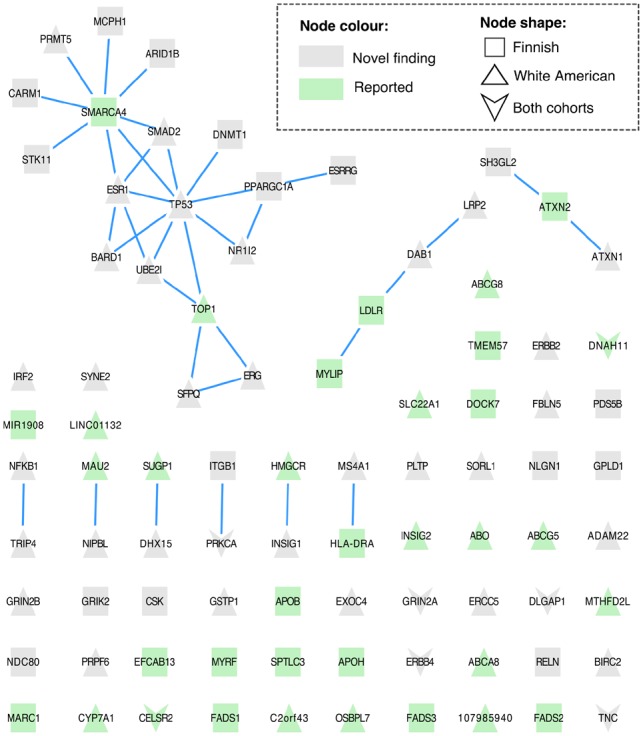
Prioritized PPI in LDL-C. This PPIN shows only interactions between prioritized genes by cNMTF. A novel finding refers to a gene either not reported or not significant in GWAS catalogue ($P<10^{-8}$)

The interaction between novel genes and seed genes in the PPIN is presented in Figure 5, Supplementary Figures S9 and S11. We mapped the results onto the PPIN and retrieved only the edges between prioritized genes. The graph for LDL-C shows well-known genes for the lipid metabolism: *LDLR*, *APOB*, *APOH* and *FADS1/FADS2/FADS3*. However, most of these genes appear isolated from other prioritized genes, which are a consequence of using only the first neighbours from the PPIN rather than the whole lipid pathways in the input.

It is also clear in the network that Finnish and white Americans have just a few prioritized genes in common (12% of the genes intersect). Indeed, some of the interactions between novel loci and key proteins were observed only in specific populations. For example, *SMARCA4-CARM1* is an interaction captured only in the Finnish cohort (the tumour-suppressive gene *CARM1*, interacts with a gene associated with abnormal LDL-C levels and required for tumour cell growth, *SMARCA4*). Similarly, in the white Americans, we noted the interaction *HMGCR-INSIG1* (the regulator of lipogenesis *INSIG1* binds an enzyme controlling the production of cholesterol, *HMGCR*). This specificity of results by population arise from variable allele frequencies and the LD pattern (Deo *et al.*, 2009).

## 4 Discussion and conclusions

We developed a data integration framework to address the problem of SNV and loci prioritization. cNMTF extracts relevant patterns of information from genotypes, phenotypes and molecular data via dimensionality reduction, finds clustering patterns and scores the associations with the phenotype. A key feature of cNMTF is performing multiple-SNV analysis by means of the SNV–SNV network. This strategy allows for the sharing of information between variants depending on their network connectivity and the similarity of genotypes across individuals.

We have provided cNMTF with capabilities to capture gene-trait associations that are not significant in the univariate studies due to insufficient statistical power. The method also unveiled well-known genes involved in lipid metabolism that have not been prioritized in the Finnish and white American cohorts, thereby, complementing current findings of LRMs.

Another relevant feature of cNMTF is the correction for detrimental effects of population stratification, which are particularly problematic when using matrix factorization. In Supplementary File S1 (Supplementary Sections S10–S12 and Figs S18–S21) we expand the discussion on how the algorithm generates solutions regardless the subjects’ ancestry, while minimizing the rate of spurious findings.

Although our study shows an implementation of cNMTF with SNV–SNV networks based on PPIs, the algorithm is suitable for studying other omic datasets in future works. The input can be modified to allow for the integration of genotypes with metabolic, transcriptomic or proteomic data by means of weighted networks. This would give insights on the genetic heterogeneity resulting from pathways, where current methods treat all the genes as equivalent and do not model their interactions (Leiserson *et al.*, 2013). In addition, cNMTF can be easily adapted to go beyond the case–control design. For example, this can be used for patient stratification and definition of tumour subtypes in cancer research, where a number of clusters could be assessed simultaneously.

With regard to the computational features of the algorithm, our research expands the field of NMTF-derived methods (non-negative matrix tri-factorization). To date, regularizations of NMTF are characterized by the use of graph Laplacian (Shang *et al.*, 2012), constrained clusters (Li, 2010), rules in matrix definition (Ding *et al.*, 2006; Gu and Zhou, 2009) and knowledge transference between input matrices (Gligorijevic *et al.*, 2016a, c). Here, we showed the regularization of NMTF via the combined use of kernels, and stated principles for adequate data weighting and confounder correction. Future work can extent these principles for the study of continuous phenotypes or the formulation of SNV scores from multi-layer omic networks.

This work has limitations in the algorithm implementation. First, the method can only be evaluated on a subset of SNVs due to the computational cost of matrix operations at the genome-wide level. We limited the analyses to disease-associated genes reported in GWAS catalogue (seeds) and the first neighbourhood of seed genes in the PPIN. Genes outside these filtering rules are lost in our study cases, so we have potentially lost disease-associated locus not interacting with our seed genes. Nonetheless, the algorithm can be tailored to explore larger sets of seed genes or indirect PPIs, depending on the scope and the computational resources available to that aim.

Second, the inclusion of PPI data could bring bias to the results. Hub genes in the protein interactome have a higher chance to interact with our set of seed genes and they are more likely to be included in the input (e.g. *TP53* connects 11% of proteins in the BioGrid network). Similarly, candidate SNVs in hub genes could be connected to more damaging SNVs. When the algorithm is executed, the diffusion of information through the network will favour the proximity between candidate and damaging variants.

To address this limitation, we introduced the normalized graph Laplacian in the objective function and analyzed the node degree of prioritized genes/SNVs in their networks (Supplementary File S1, Section S4.1 and Fig. S17). However, no significant differences in the median node degree of prioritized genes/SNVs were observed between settings of the algorithm (basic Laplacian versus normalized Laplacian, Wilcoxon test, *P *>* *0.24). Please note that we are already performing a normalization in the edges of the SNV–SNV network to remove bias for genes harbouring multiple variants. Therefore, these results indicate that hub genes/SNVs could be prioritized because they may play a relevant role in the lipid processes and should be integrated in the analyses (e.g. *APOB* is a key hub gene connecting 12 proteins). We conclude that the connectivity of the network itself cannot prioritize variants unless they show moderate association signals in the genotype data.

Another limitation of cNMTF is that some genes associated with the lipid trait (e.g. *CELSR2, LPL*) were not prioritized. This is due to the clustering nature of the method, the different sources of information contributing to the final results and our still limited knowledge of the human interactome. Particularly, the SNV–SNV network leads to strong association signals boosting the weak variants; however, the opposite can also occur (the strong association is masked) if clustering patterns are not observed in the genotype data, or the network is saturated of very poor associations. Consequently, we see our method as a complementary tool for single-SNV studies because its performance depends on the clusters and the connectivity of weak–strong variants.

In conclusion, we have presented cNMTF as an alternative approach to prioritize variants and genes for follow-up studies. Given the satisfactory results with lipid traits and the flexibility of cNMTF to handle interrelated but disparate sources of data, this study provides valuable guidelines for future integrative approaches in the field.

## Funding

Luis G. Leal is supported by the President’s PhD Scholarship Scheme from Imperial College London. Alessia David is supported by the Wellcome Trust (grant WT/104955/Z/14/Z). Clive Hoggart is supported by the European Union’s Horizon 2020 research and innovation programme (grant 668303). The NFBC1966 received financial support from the Academy of Finland (project grants 104781, 120315, 129269, 1114194, 24300796), University Hospital Oulu, Biocenter, University of Oulu, Finland (75617), National Heart, Lung and Blood Institute (5R01HL087679-02) through the STAMPEED program (1RL1MH083268-01), National Institutes of Health/The National Institute of Mental Health (5R01MH63706: 02) and the Medical Research Council, UK (MR/M013138/1). The program is currently being funded by the DynaHEALTH action (H2020-633595) and academy of Finland EGEA-project (285547). The DNA extractions, sample quality controls, biobank up-keeping and aliquoting were performed in the National Public Health Institute, Biomedicum Helsinki, Finland and supported financially by the Academy of Finland and Biocentrum Helsinki. The eMERGE Network was initiated and funded by the National Human Genome Research Institute, in conjunction with additional funding from National Institute of General Medical Sciences through the following grants: (U01-HG-004610) Group Health Cooperative/University of Washington; (U01-HG-004608) Marshfield Clinic Research Foundation and Vanderbilt University Medical Center; (U01-HG-04599) Mayo Clinic; (U01HG004609) Northwestern University; (U01-HG-04603) Vanderbilt University Medical Center, also serving as the Administrative Coordinating Center; (U01HG004438) Center for Inherited Disease Research and (U01HG004424) the Broad Institute serving as Genotyping Centers.

*Conflict of Interest*: none declared.

## Supplementary Material

btz310_Supplementary_Data
