# Supplementary material for: Identification of disease-associated loci using machine learning for genotype and network data integration
Source: Bioinformatics. 2019 May 9;35(24):5182–90. doi: 10.1093/bioinformatics/btz310 (PMC6954643; doi:10.1093/bioinformatics/btz310)
Supplement: btz310_Supplementary_Data [file btz310_supplementary_data.zip › btz310-Suppl_data/Supplementary_File_S1.pdf]

Supplementary File S1

Identification of disease-associated loci using machine learning for genotype and network data integration.

Luis G. Leal[1*], Alessia David[1], Marjo-Riita Jarvelin[2,3,4,5,6], Sylvain Sebert[2,3], Minna Ruddock[2], Ville Karhunen[2,3,4,5,6], Eleanor Seaby[7], Clive Hoggart[8], Michael J.E. Sternberg[1*]

1 Department of Life Sciences, Centre for Integrative Systems Biology and Bioinformatics, Imperial College London, London, United Kingdom

2 Center for Life Course Health Research, Faculty of Medicine, University of Oulu, Oulu, Finland

3 Biocenter Oulu, University of Oulu, Oulu, Finland

4 Unit of Primary Health Care, Oulu University Hospital, Oulu, Finland

5 Department of Epidemiology and Biostatistics, MRC-PHE Centre for Environment and Health, School of Public Health, Imperial College London, London, United Kingdom

6 Department of Life Sciences, College of Health and Life Sciences, Brunel University London, London, United Kingdom

7 Program in Medical and Population Genetics, Broad Institute of MIT and Harvard, Cambridge, MA, USA

8 Department of Medicine, Imperial College London, London, United Kingdom

* Corresponding authors: lgl15@imperial.ac.uk, m.sternberg@imperial.ac.uk

---

Contents

S1 Datsets **6**

S2 Simulating GWAS data with population structures **6**

S3 Preprocessing the phenotype **8**

S3.1 Correcting for confounders other than population origin 8

S3.2 Binarising the continuous phenotype 9

S4 Minimising the objective function **9**

S4.1 Update rules 9
S4.2 Iterations 12

S5 On the uniqueness of cNMTF solutions **13**

S6 Consensus solutions **13**

S7 Optimal number of clusters of SNVs **14**

S8 Grid search of penalization parameters **15**

S9 Weighting the information transferred from penalization terms **15**
S9.1 Information transferred into U 16
S9.2 Information transferred into V 17

S10 Correcting for population structures in LRMs **18**

S11 Performance of cNMTF under population structures **19**

S12 Correcting population structures in simulated GWAS data **19**

List of Tables

S1 Metabolic traits and confounder variables in the cohorts. 22
S2 Classification of lipoproteins and triglycerides levels by NIH. 22
S3 Coefficients in a multiple regression model for each trait 23
S4 Optimal cNMTF parameters 24
S5 Enriched biological annotations in the prioritised genes. 24
S6 Configuration of synthetic data with population structures. 25
S7 Clustering of white Americans from eMERGE and 1,000 Genomes Project data . . . 25

List of Figures

S1 Construction of the SNV-SNV network. 26
S2 Consensus clustering and consensus SNV score. 27

S3 Distribution of the delta SNV score for LDL-C in the Finnish cohort 30
S4 Weighting the information contribution of the genotyping data and the prior knowledge in the final results. 31
S5 Selection of optimal parameters 32
S6 Weighting the network contribution in the final results. 33
S7 Dispersion of delta scores for LDL-C 34
S8 Prioritised genes in HDL-C. 34
S9 Prioritised protein protein interactions in HDL-C. 35
S10 Prioritised genes in TG. 36
S11 Prioritised protein protein interactions in TG. 36
S12 Impact of prioritised variants 37
S13 Genes in the input and results of cNMTF 37
S14 Dispersion of prioritised genes in cNMTF vs LRM 38
S15 Prioritising lipid-associated genes from GWAS catalog 39
S16 Basic graph Laplacian versus Normalised graph Laplacian 40
S17 Changes in gene prioritisation when clumping SNVs in high LD 41
S18 Evaluating the confounding effect of population structures in the clustering results . . 42
S19 Correcting population structures in LRM and cNMTF 43
S20 Confounding effect of population structures 44
S21 PCA of synthetic GWAS data with population structures. 45

Nomenclature

α Significance level

β Regression coefficients vector

β_0 Intercept in a regression model

$\cdot_F^2$ Frobenius norm

$\Delta\Omega$ Vector of delta SNV scores.

ϵ Minimum relative change in J

$\gamma_1, \gamma_2, \gamma_3$ Penalization parameters

λ_{GC} Genomic control factor

$\boldsymbol{\Omega}$ Consensus SNV score matrix

$\overline{\boldsymbol{C}}_{\boldsymbol{R}}$ Consensus relationship matrix

$\overline{\boldsymbol{C}}_{\boldsymbol{U}}$ Consensus connectivity matrix of SNVs

$\overline{\boldsymbol{C}}_{\boldsymbol{V}}$ Consensus connectivity matrix of subjects

$\boldsymbol{A}$ Kernel matrix generated by the linear kernel a on the population origin information.

$\boldsymbol{C_{U,t}}$ Binary connectivity matrix of SNVs

$\boldsymbol{C_{V,t}}$ Binary connectivity matrix of subjects

$\boldsymbol{D_U}$ Diagonal degree matrix of $\boldsymbol{W_U}$

$\boldsymbol{K}$ Kernel matrix generated by the linear kernel k on the clustering information.

$\boldsymbol{L_U}$ Laplacian matrix of a SNV-SNV network

$\boldsymbol{R}$ Relationship matrix for the genotyping data

$\boldsymbol{S}$ Relationship matrix for clusters of SNVs and subjects.

$\boldsymbol{U}^*$ Matrix encoding the maximum entries in the rows of $\boldsymbol{U}$

$\boldsymbol{U}$ Cluster indicator matrix for SNVs

$\boldsymbol{V}^*$ Matrix encoding the maximum entries in the rows of $\boldsymbol{V}$

$\boldsymbol{V_o}$ Phenotype matrix for the phenotyping data.

$\boldsymbol{V}$ Cluster indicator matrix for subjects

$\boldsymbol{W_U}$ Weighted adjacency matrix of the SNV-SNV network

$\boldsymbol{X}$ Matrix of explanatory variables

$\boldsymbol{z}_a$ Population labels

$\boldsymbol{z}_o$ Phenotype labels

$\boldsymbol{z}_v$ Cluster membership of subjects

$\mathcal{F}, \mathcal{G}$ Feature spaces of random variabes X and Y

ρ_{k_1} Dispersion coefficient for the clusters of SNVs

ε Residuals in a regression model

$\varepsilon_{lower}, \varepsilon_{upper}$ Residual cut-off points

ζ_1, ζ_2 Parameters of the beta distribution

d Total node degree across clusters

$d_{U,i}$ Total node degree of the SNVs in the i-th cluster

F_{ST} Allele frequency variation within the population

$G_{i,j}$ Genotype of subject j in the SNV i

J Objective function

k_1 Number of clusters of SNVs

k_2 Number of clusters of subjects

m Number of subjects

n Number of variants

NMI Normalized Mutual Information

p_s^* Ancestral allele frequency

P_s Distribution of allele frequencies

$p_{s,l}$ Allele frequency of allele s in population l

Q Maximum number of iterations for convergency of the algorithm

RR Relative risk

T Number of repetitions of the algorithm

$w_R, w_{L_u}, w_{V_o}, w_A$ Weighted information contribution from genotyping data and prior knowledge

X_s Genotypes observed at loci s

Y Random variable denoting the observed trait

Y_{lower}, Y_{upper} Lower and upper cut-off points for the trait

AIC Akaike Information Criterion

BMI Body Mass Index

BMIB Body Mass Index at birth

cNMTF Corrected Non-negative matrix tri-factorization

GWAS Genome-wide Association Studies

HDL-C High-density lipoproteins cholesterol

LDL-C Low-density lipoproteins cholesterol

NFBC The Northern Finland Birth Cohort

NIH National Institutes of Health

NMTF Non-negative matrix tri-factorization

PCA Principal Components Analysis

RMs Regression models

SNVs Single Nucleotide Variants

SVMs Support Vector Machines

TG Triglycerides

S1 Datsets

The NFBC data set

A total of 329,091 SNVs genotyped in 5,402 samples of subjects were accessed from The Northern Finland Birth Cohort 1966 (NFBC). The NFBC is characterized by subjects born in the same year and with homogeneous genetic background. In the preprocessing of the data, subjects not fasting or having diabetic medication were removed. Subjects' lipid profile was obtained on fasting samples. There is no evidence of any treatment with antilipemic medications in these subjects because they are young adults of the same age (31 years old).

The eMERGE data set

A cohort of American subjects of self-reported white ethnicity was obtained from the eMERGE network (dbGaP Study Accession: phs000360.v3.p1). This cohort consists of 561,490 SNVs from 6,100 subjects spanning a larger age range and heterogeneity than the NFBC cohort (Table 1, main document). The lipid measurements were obtained from the Electronic Medical Records of a longitudinal panel, as reported by the Northwestern University (dbGaP Document Accession: phd003541.1). This procedure guarantees that the lipid measurements are not biased by antilipemic drugs, hormone replacement therapies, type 2 diabetes medications or comorbidities (*e.g.*, cancer, hypothyroidism).

S2 Simulating GWAS data with population structures

We generated genetic data with discrete populations structures following the methodology of Price *et al.* (2006) [1]. In this simulation setting we described a synthetic SNV in terms of the allele frequency, and the relative risk, RR. The allele frequency tells whether a SNV is more prevalent in any of the populations; the relative risk tells whether a SNV is associated or not with the phenotype [2, 3].

The first step to simulate a GWAS dataset is to select the allele frequency, $p_{s,l}$, for the allele s in the population l. This frequency can be fixed to make the SNV more prevalent in any of the populations (*e.g,* $p_{s,1} = 0.10$ and $p_{s,2} = 0.40$ refers to a population-dependent SNV). The frequency can also be randomly draw to make the frequencies just slightly different between populations (*e.g,* $p_{s,1} = 0.35$ and $p_{s,2} = 0.36$ refers to a population-independent SNV). The latter option makes use of the beta distribution, $P_s \sim Beta(\zeta_1, \zeta_2)$, with parameters $\zeta_1 = p_s^*(1 - F_{ST})/F_{ST}$ and $\zeta_2 = (1 -$

$p_s^*)(1 - F_{ST})/F_{ST}$. Where F_{ST} is an standardized measure of allele frequency variation within the population ($F_{ST} = 0.01$) and p_s^* is the ancestral allele frequency drawn from an uniform distribution $P_s^* \sim Unif(0.1, 0.5)$.

Once the allele frequency is fitted, this is used to generate the genotypes for individuals. It is assumed that genotypes (*i.e, AA, AB,* or *BB*) relate to phenotypes (*i.e*, case or control) via a genetic multiplicative model, where the causal allele has a genotype relative risk. If we fit $RR > 1$ then the SNV has signal of association with the phenotype. If we fit $RR = 1$ the SNV has no association with the phenotype:

Relative frequencies per genotype and phenotype (case or control) in a multiplicative genetic model

**Phenotype**	***AA***	***AB***	***BB***
**Controls**	$(1 - p_s)^2$	$2p_s(1 - p_s)$	p_s^2
**Cases**	$(1-p_s)^2/t$	$2(RR)p_s(1 - p_s)/t$	$RR^2p_s^2/t$

The frequency of cases is rescaled to sum up to 1 by $t = (1 - p_s)^2 + 2(RR)p_s(1 - p_s) + (RR)^2p_s^2$.

As stated above, a synthetic SNV can be described by combinations of allele frequencies and genetic risks; consequently, there are four types of synthetic SNVs:

- **Type I**: Population-independent SNVs without association with the phenotype ($P_s \sim Beta(\zeta_1, \zeta_2)$, $RR = 1$).
- **Type II**: Population-dependent SNVs without association with the phenotype ($P_{s,1} = 0.1, P_{s,2} = 0.4$, $RR = 1$).
- **Type III**: Population-independent SNVs with association with the phenotype ($P_s \sim Beta(\zeta_1, \zeta_2)$, $RR = 3$).
- **Type IV**: Population-dependent SNVs with association with the phenotype ($P_{s,1} = 0.1, P_{s,2} = 0.4$, $RR = 3$).

We simulated five datasets containing different combinations of synthetic SNVs (Table S6). The datasets comprised 2 populations, 10,000 SNVs, 1,000 cases and 1,000 controls (40% cases in population 1 and 60% cases in population 2).

S3 Preprocessing the phenotype

S3.1 Correcting for confounders other than population origin

The traits of interest were adjusted by following the same protocol of Sabatti *et. al.,* [4]. First, we removed subjects with unknown data and preprocessed each confounder variable as follows:

- The pregnancy status, use of oral contraceptives and sex were reduced to a single variable, SOCPG, that has one level for males and four levels for females corresponding to the combinations of pregnancy (yes, no) and contraceptives (yes, no).
- The smoking habits takes two levels (yes, no) depending on whether the subject has ever smoked.
- The alcohol consumption takes continuous levels starting at 0 grams per day.
- The BMI was categorised by placing the observed values in five quantiles. Thus, we used a categorical variable of five levels in the analysis. Same procedure was applied to the variable age.
- The BMI at birth (BMIB) was initially corrected for other confounders. We regressed the BMIB on mother's smoking status, mother's weight and height, mother's parity, subject's gestational age ($>$ 37 weeks, $\leq$ 37 weeks) and sex. The residuals of this model were also categorised into five quantiles to conform the final BMIB variable.

These variables were put into a multiple regression model:

$$\boldsymbol{Y} = \boldsymbol{\beta_0} + \boldsymbol{\beta}\boldsymbol{X} + \boldsymbol{\varepsilon} \tag{S1}$$

Where the continuous trait Y is explained in terms of an intercept β_0, a matrix of confounders $\boldsymbol{X}$, a vector of regression coefficients $\boldsymbol{\beta}$, and the residuals, ε. The best model was fitted using the AIC measure in a step-wise backwards approach. Those confounders that better explain the trait and their coefficients are presented in Table S3. Note that only a few confounders, including age, smoking habits, alcohol consumption, sex and BMI were available for the white American cohort.

S3.2 Binarising the continuous phenotype

The continuous traits were binarised by labelling the subjects as *cases* or *controls*. We used the NIH's risk level categories to define lower and upper cut-off points at each trait (Y_{lower}, Y_{upper}) (Table S2) . As we are analysing the residuals from equation S1 rather than the continuous trait levels, we must translate those cut-off points (Y_{lower}, Y_{upper}) to the residuals scale. This is achieved by subtracting the intercept, β_0, from Y_{lower} and Y_{upper}.

$$\begin{aligned} \varepsilon_{lower} &= Y_{lower} - \beta_0 \\ \varepsilon_{upper} &= Y_{upper} - \beta_0 \end{aligned} \tag{S2}$$

Afterwards, we used the residual cut-off points (ε_{lower}, ε_{upper}) to label subjects. For example, in LDL, subjects with residuals $\varepsilon \leq \varepsilon_{lower}$ were put under the label *cases*, subjects with $\varepsilon \geq \varepsilon_{upper}$ were labelled as *controls* and subjects with $\varepsilon_{lower} < \varepsilon < \varepsilon_{upper}$ were removed from analysis.

The same cut-off points were applied indistintly to all subjects, which equates to different cut-off in the continuous trait values.

S4 Minimising the objective function

S4.1 Update rules

The optimization problem of cNMTF was defined in equation (2) of the main document. To solve this optimization problem and obtain locally optimal solutions of $\boldsymbol{U}$, $\boldsymbol{S}$ and $\boldsymbol{V}$, we must formulate iterative update rules based on the gradient descent algorithm [5].

First, the objective function is rewritten using the definition of the Fobrenius norm and the trace of the matrix:

$$\begin{aligned} \min_{U\geq 0, S\geq 0, V\geq 0} J_{cNMTF} = \|\boldsymbol{R} - \boldsymbol{USV}^T\|_F^2 + \gamma_1 \cdot tr(\boldsymbol{U^T L_U U}) + \gamma_2 \cdot \|\boldsymbol{V} - \boldsymbol{V_o}\|_F^2 \\ + \gamma_3 \cdot tr(\boldsymbol{VV^T HAH}) \end{aligned} \tag{S3}$$

$$\min_{U\geq 0,S\geq 0,V\geq 0} J_{cNMTF} = tr(\boldsymbol{R}\boldsymbol{R}^{T}) - 2\cdot tr(\boldsymbol{R}\boldsymbol{V}\boldsymbol{S}^{\boldsymbol{T}}\boldsymbol{U}) + tr(\boldsymbol{U}\boldsymbol{S}\boldsymbol{V}^{\boldsymbol{T}}\boldsymbol{V}\boldsymbol{S}^{\boldsymbol{T}}\boldsymbol{U}^{\boldsymbol{T}})$$
$$+\gamma_1\cdot tr(\boldsymbol{U}^{\boldsymbol{T}}\boldsymbol{L}_{\boldsymbol{U}}\boldsymbol{U})$$
$$+\gamma_2\cdot (tr(\boldsymbol{V}\boldsymbol{V}^{\boldsymbol{T}}) - 2tr(\boldsymbol{V}\boldsymbol{V}_{\boldsymbol{o}}^{\boldsymbol{T}}) + \boldsymbol{V}_{\boldsymbol{o}}\boldsymbol{V}_{\boldsymbol{o}}^{\boldsymbol{T}}))$$
$$+\gamma_3\cdot tr(\boldsymbol{V}\boldsymbol{V}^{\boldsymbol{T}}\boldsymbol{H}\boldsymbol{A}\boldsymbol{H}) \tag{S4}$$

We introduce Lagrange multipliers $\boldsymbol{\Phi}$, $\boldsymbol{\Xi}$, $\boldsymbol{\Theta}$, for $\boldsymbol{U}$, $\boldsymbol{S}$ and $\boldsymbol{V}$, respectively, and define a Lagrange function:

$$\mathcal{L} = tr(\boldsymbol{R}\boldsymbol{R}^{T}) - 2\cdot tr(\boldsymbol{R}\boldsymbol{V}\boldsymbol{S}^{\boldsymbol{T}}\boldsymbol{U}) + tr(\boldsymbol{U}\boldsymbol{S}\boldsymbol{V}^{\boldsymbol{T}}\boldsymbol{V}\boldsymbol{S}^{\boldsymbol{T}}\boldsymbol{U}^{\boldsymbol{T}})$$
$$+\gamma_1\cdot tr(\boldsymbol{U}^{\boldsymbol{T}}\boldsymbol{L}_{\boldsymbol{U}}\boldsymbol{U})$$
$$+\gamma_2\cdot (tr(\boldsymbol{V}\boldsymbol{V}^{\boldsymbol{T}}) - 2tr(\boldsymbol{V}\boldsymbol{V}_{\boldsymbol{o}}^{\boldsymbol{T}}) + \boldsymbol{V}_{\boldsymbol{o}}\boldsymbol{V}_{\boldsymbol{o}}^{\boldsymbol{T}})) \tag{S5}$$
$$+\gamma_3\cdot tr(\boldsymbol{V}\boldsymbol{V}^{\boldsymbol{T}}\boldsymbol{H}\boldsymbol{A}\boldsymbol{H})$$
$$+tr(\boldsymbol{\Phi}\boldsymbol{U}^{\boldsymbol{T}}) + tr(\boldsymbol{\Theta}\boldsymbol{V}^{\boldsymbol{T}}) + tr(\boldsymbol{\Xi}\boldsymbol{S}^{\boldsymbol{T}})$$

Where the Lagrange multipliers are $\boldsymbol{\Phi} = [\phi_{i,j}] \in \Re^{k_2,n}$, $\boldsymbol{\Xi} = [\xi_{i,j}] \in \Re^{k_1,k_2}$ and $\boldsymbol{\Theta} = [\theta_{i,j}] \in \Re^{k_1,m}$.

Now the derivatives of the Lagrange function are taken with respect to $\boldsymbol{U}$, $\boldsymbol{S}$ and $\boldsymbol{V}$:

$$\frac{\partial\mathcal{L}}{\partial U} = -2\cdot \boldsymbol{R}\boldsymbol{V}\boldsymbol{S}^{\boldsymbol{T}} + 2\cdot \boldsymbol{U}\boldsymbol{S}\boldsymbol{V}^{\boldsymbol{T}}\boldsymbol{V}\boldsymbol{S}^{\boldsymbol{T}} + 2\cdot\gamma_1\boldsymbol{L}_{\boldsymbol{U}}\boldsymbol{U} + \boldsymbol{\Phi} \tag{S6}$$

$$\frac{\partial\mathcal{L}}{\partial S} = -2\cdot \boldsymbol{U}^{\boldsymbol{T}}\boldsymbol{R}\boldsymbol{V} + 2\cdot \boldsymbol{U}^{\boldsymbol{T}}\boldsymbol{U}\boldsymbol{S}\boldsymbol{V}^{\boldsymbol{T}}\boldsymbol{T} + \boldsymbol{\Xi} \tag{S7}$$

$$\frac{\partial\mathcal{L}}{\partial V} = -2\cdot \boldsymbol{R}^{\boldsymbol{T}}\boldsymbol{U}\boldsymbol{S} + 2\cdot \boldsymbol{V}\boldsymbol{S}^{\boldsymbol{T}}\boldsymbol{U}^{\boldsymbol{T}}\boldsymbol{U}\boldsymbol{S} + 2\cdot\gamma_2(\boldsymbol{V} - \boldsymbol{V}_{\boldsymbol{o}}) + \gamma_3(\boldsymbol{H}\boldsymbol{A}\boldsymbol{H}\boldsymbol{V} + (\boldsymbol{H}\boldsymbol{A}\boldsymbol{H})^{T}V) + \boldsymbol{\Theta} \tag{S8}$$

Then, the Karush-Kuhn-Tucker (KKT) conditions are declared as follows:

$$\phi_{i,j}\boldsymbol{U}_{i,j} = 0 \tag{S9}$$

$$\xi_{i,j}\boldsymbol{S}_{i,j} = 0 \tag{S10}$$

$$\theta_{i,j}\boldsymbol{V}_{i,j} = 0 \tag{S11}$$

After setting the derivatives to zero, we obtain the Lagrange multipliers:

$$\boldsymbol{\Phi} = 2[\boldsymbol{RVS^T} - \boldsymbol{USV^TVS^T} - \gamma_1\boldsymbol{L_U U}] \tag{S12}$$

$$\boldsymbol{\Xi} = 2[-\boldsymbol{U^TRV} - \boldsymbol{U^TUSV^TT}] \tag{S13}$$

$$\boldsymbol{\Theta} = 2[\boldsymbol{R^TUS} - \boldsymbol{VS^TU^TUS} - \gamma_2(\boldsymbol{V} - \boldsymbol{V_o})] - \gamma_3(\boldsymbol{HAHV} + (\boldsymbol{HAH})^T V) \tag{S14}$$

Finally, we formulate the update rules by using the KKT conditions mentioned above and the gradient descent algorithm [5]:

$$\boldsymbol{U}_{i,j} \leftarrow \boldsymbol{U}_{i,j}\frac{[\boldsymbol{RVS}^T + \gamma_1\boldsymbol{W_U U}]_{i,j}}{[\boldsymbol{USV^TVS^T} + \gamma_1\boldsymbol{D_U U}]_{i,j}} \tag{S15}$$

$$\boldsymbol{S}_{i,j} \leftarrow \boldsymbol{S}_{i,j} \frac{[\boldsymbol{U}^{\boldsymbol{T}}\boldsymbol{R}\boldsymbol{V}]_{i,j}}{[\boldsymbol{U}^{\boldsymbol{T}}\boldsymbol{U}\boldsymbol{S}\boldsymbol{V}^{\boldsymbol{T}}\boldsymbol{V}]_{j,l}} \tag{S16}$$

$$\boldsymbol{V}_{i,j} \leftarrow \boldsymbol{V}_{i,j} \frac{[\boldsymbol{R}^{\boldsymbol{T}}\boldsymbol{U}S + \gamma_2 \boldsymbol{V_o}]_{i,j}}{[\boldsymbol{V}\boldsymbol{S}^{\boldsymbol{T}}\boldsymbol{U}^{\boldsymbol{T}}\boldsymbol{U}\boldsymbol{S} + \gamma_3 \boldsymbol{H}\boldsymbol{A}\boldsymbol{H}\boldsymbol{V} + \gamma_2 \boldsymbol{V}]_{i,j}} \tag{S17}$$

Here, the entries of matrices ($\boldsymbol{U}_{i,j}$, $\boldsymbol{S}_{i,j}$ and $\boldsymbol{V}_{i,j}$ are updated iteratively when multiplying their values at a given iteration by the ratio of negative and positive terms in equations S12, S13 and S14. We have also used two definitions of the Laplacian matrix:

- **Basic graph Laplacian**: $\boldsymbol{L_U} = \boldsymbol{D_U} - \boldsymbol{W_U}$, where $\boldsymbol{W_U}$ is the weighted adjacency matrix of the SNV-SNV network, and $\boldsymbol{D_U}$ is the diagonal degree matrix of $\boldsymbol{W_U}$.
- **Normalised graph Laplacian**: $\boldsymbol{L_U} = \boldsymbol{I} - \boldsymbol{D_U}^{-\frac{1}{2}} \boldsymbol{W_U} \boldsymbol{D_U}^{-\frac{1}{2}}$, where $\boldsymbol{I}$ is the identity matrix of size $n \times n$ with ones on the main diagonal.

For the Nomalised graph Laplacian the update rule of $\boldsymbol{U}$ changes to:

$$\boldsymbol{U}_{i,j} \leftarrow \boldsymbol{U}_{i,j} \frac{[\boldsymbol{R}\boldsymbol{V}\boldsymbol{S}^T + \gamma_1 \boldsymbol{D}_{\boldsymbol{U}}^{-\frac{1}{2}} \boldsymbol{W_U} \boldsymbol{D}_{\boldsymbol{U}}^{-\frac{1}{2}} \boldsymbol{U}]_{i,j}}{[\boldsymbol{U}\boldsymbol{S}\boldsymbol{V}^{\boldsymbol{T}}\boldsymbol{V}\boldsymbol{S}^{\boldsymbol{T}} + \gamma_1 \boldsymbol{I}\boldsymbol{U}]_{i,j}} \tag{S18}$$

S4.2 Iterations

Initialisation of those matrices can be done randomly or following a Single Value Decomposition based approach [6]. Here both initialisation approaches were tested but we did not find differences in our results.

To terminate the iterations, any of the following criteria must be fulfilled: i) The maximum number of iterations is reached ($Q = 500$), ii) The minimum relative change in the objective function is reached $|J_q - J_{q-1}| > \epsilon$, $\epsilon = 1e - 5$ with $q = 1, \ldots Q$. (See Algorithm 1)

S5 On the uniqueness of cNMTF solutions

The cNMTF algorithm, like other matrix factorisations, generates approximate solutions and its low-rank matrices are in general non-unique. It means that any solution of $\boldsymbol{U}$ $\boldsymbol{S}$ and $\boldsymbol{V}$ is not the only interpretation of the data in $\boldsymbol{R}$, $\boldsymbol{W_u}$ and $\boldsymbol{V_o}$. However, relevant patterns can be retrieved by running the algorithm multiple times with different initialisations, extracting the clustering solutions from each repetition and combining these results to conform a consensus solution.

At each repetition of the algorithm, the solutions of $\boldsymbol{U}$ $\boldsymbol{S}$ and $\boldsymbol{V}$ are summarised in consensus matrices that we explore later to find clusters and prioritise variants (Figure S2). We observed that $T = 100$ repetitions of the algorithm are enough to reach unique solutions and robust prioritisations for the different traits.

Algorithm 1 cNMTF. See Figure S2 for details.

Input: $\boldsymbol{R}$, $\boldsymbol{W_u}$, $\boldsymbol{V_o}$, $\boldsymbol{H}$, $\boldsymbol{A}$, k_i, k_j, γ_1, γ_2, γ_3, Q, ϵ, T.
Output: Subject and SNV cluster membership, delta SNV scores, $\varDelta\varOmega_s$.

while $t \leq T$ **do**
  Initialize the entries of $\boldsymbol{U}_t$, $\boldsymbol{S}_t$ and $\boldsymbol{V}_t$
  while $q \leq Q$ **and** $|J_q - J_{q-1}| > \epsilon$ **do**
    Update entries of $\boldsymbol{S}_t$ with: $S_{j,l} \leftarrow S_{j,l} \frac{[U^T R V]_{j,l}}{[U^T U S V^T V]_{j,l}}$
    Update entries of $\boldsymbol{U}_t$ with:
    $U_{i,j} \leftarrow U_{i,j} \frac{[R V S^T + \gamma_1 W_U U]_{i,j}}{[U S V^T V S^T]_{i,j} + \gamma_1 D_U U]_{i,j}}$
    Update entries of $\boldsymbol{V}_t$ with:
    $V_{k,j} \leftarrow V_{k,j} \frac{[R^T U S + \gamma_2 V_o]_{k,j}}{[V S^T U^T U S + \gamma_3 H A H V + \gamma_2 V]_{k,j}}$
  end while
  Extract the clustering membership of subjects and SNVs.
  Compute the SNV score matrix, $\boldsymbol{\varOmega_t}$.
end while
Find consensus clustering of subjects and SNVs.
Calculate the consensus score matrix, $\boldsymbol{\varOmega}$.
Calculate the delta score vector, $\varDelta\varOmega_s$.

S6 Consensus solutions

Consensus clustering

In Algorithm 1, each optimisation solution t produces a $\boldsymbol{V_t}$ matrix which is used for hard clustering of subjects by finding the maximum row entries. Thus, each subject belongs to one cluster as the

matrix $\boldsymbol{V_t}$ is binarised to $\boldsymbol{V}^*$, where $V_t^*[i,j] = 1$ if $V_t[i,j] = \max(V_t[,j])$. Then, the cluster assignments are collected in a binary connectivity matrix, $\boldsymbol{C_{V,t}}$, of size $m \times m$ with entry $C_{V,t}[i,l] = 1$ if subjects i and l belong to the same cluster, and $C_{V,t}[i,l] = 0$ otherwise. The connectivity matrices are averaged across T repetitions of the algorithm in the consensus connectivity matrix $\overline{\boldsymbol{C}}_{\boldsymbol{V}}$. This consensus matrix has entries ranging from 0 to 1 and represent how often we observe two subjects in the same cluster across repetitions of the algorithm. Finally, we apply average hierarchical clustering on the off-diagonal entries of $\overline{\boldsymbol{C}}_{\boldsymbol{V}}$ to find the consensus clustering of subjects [7]. Same procedure is established for the consensus clustering of SNVs in Figure S2.

A consensus score to prioritise SNVs

Prioritisation is based on the relative importance of SNVs between clusters of subject. At each repetition of the algorithm, we compute the product of $\boldsymbol{U_t}$ and $\boldsymbol{S_t}$ to generate a score matrix $\boldsymbol{\Omega_{t}}_{(n\times k_2)}$. This matrix summarizes the effect of single SNVs on clusters of subjects with specific phenotypes and can be used to prioritise the SNVs. However, due to the non-uniqueness of results, cluster assignments change through repetitions of the algorithm and we must consider a consensus approach as well. Such consensus is achieved by averaging the product of $\boldsymbol{\Omega_t}$ and $\boldsymbol{V}^*$ through repetitions; so we conform a consensus relationship matrix $\overline{\boldsymbol{C}}_{\boldsymbol{R}}$ of size $n \times m$. As a last step, we compute our consensus score matrix $\boldsymbol{\Omega}_{(n\times k_2)}$ by assessing the median entries of $\overline{\boldsymbol{C}}_{\boldsymbol{R}}$ among the consensus clusters of subjects.

Complexity analysis

The computational runtime of the algorithm is:
$O\left(\max\{k_1, k_2\}QTnm + (k_2QT + n)m^2 + m^3 + Tn^2\right)$. Hence, the complexity is a function of the number of variants n, number of subjects m, number of clusters k_1 and k_2, the number of iterations till convergence Q, and the number of repetitions to find consensus solutions, T. If $n \gg m \gg T \gg Q \gg k_1 \gg k_2$, the computational complexity is quadratic in the number of variants, $O(n^2)$.

S7 Optimal number of clusters of SNVs

We used a dispersion coefficient, ρ_{k_1}, to summarize the consistency of clustering assignments throughout repetitions of the algorithm [**?**]. When cluster assignments are stable, the entries of a SNV con-

sensus connectivity matrix, $\overline{\mathbf{C_U}}$, will be either close to 1 or 0 (See S2 for the definition of $\overline{\mathbf{C_U}}$). The optimal ρ_{k_1} for $k_1 : \{5, 10, 15, 20, 30, 50\}$ is identified in Figure S5A.

S8 Grid search of penalization parameters

The selection of parameters γ_1, γ_2 and γ_3 is based on a grid search. We tested parameters from 0 to a maximum value which must guarantee convergence of J. Thus, the upper limit is a value that guarantees same order of magnitude among terms in the objective function. The range of values for each parameter are:

$$\gamma_1 \in \left[0,\ \frac{\|\boldsymbol{R} - \boldsymbol{U}\boldsymbol{S}\boldsymbol{V}^T\|_F^2}{tr\left(\boldsymbol{U}^T \boldsymbol{L_U} \boldsymbol{U}\right)}\right] \tag{S19}$$

$$\gamma_2 \in \left[0,\ \frac{\|\boldsymbol{R} - \boldsymbol{U}\boldsymbol{S}\boldsymbol{V}^T\|_F^2}{tr\left(\boldsymbol{V}\boldsymbol{V}^{\boldsymbol{T}} \boldsymbol{H}\boldsymbol{A}\boldsymbol{H}\right)}\right] \tag{S20}$$

$$\gamma_3 \in \left[0,\ \frac{\|\boldsymbol{R} - \boldsymbol{U}\boldsymbol{S}\boldsymbol{V}^T\|_F^2}{\|\boldsymbol{V} - \boldsymbol{V_o}\|_F^2}\right] \tag{S21}$$

These limits would avoid convergence problems in J because the penalization terms are not bigger than the main factorization term.

Afterwards, we chose grid values for $\gamma_1 : \{0, 0.001\gamma_{1\,\max}, 0.1\gamma_{1,\max}, \gamma_{1,\max}\}$, and similarly for γ_2 and γ_3.

S9 Weighting the information transferred from penalization terms

To weight the effect of penalization terms ($\boldsymbol{L_u}$, $\boldsymbol{A}$ and $\boldsymbol{V_o}$) in our results, we selected variables that tell how much information is transferred into $\boldsymbol{U}$ and $\boldsymbol{V}$ when γ_1, γ_2 and γ_3 increase (Figure S4).

S9.1 Information transferred into $\boldsymbol{U}$

We use γ_1 to trade-off the penalization from the SNV-SNV network ($\boldsymbol{L_u}$). The penalization term forces connected SNVs to belong to the same cluster in $\boldsymbol{U}$, so highly connected communities in the network are more likely to be clustered together. Any change in $\boldsymbol{U}$ as a result of γ_1 is measured with the total node degree within clusters, d. This variable quantifies the number of edges observed within the SNV clusters:

$$d = \sum_{i=1}^{k_1} d_{U,i} \tag{S22}$$

Where $d_{U,i}$ is the total node degree of the SNVs in the i-th cluster.

If the penalization is removed ($\gamma_1 = 0$), the clusters only capture SNVs with similar row-vectors in $\boldsymbol{R}$ (Fig 3A, main paper). Thus, the information transferred from $\boldsymbol{L_u}$ is null ($w_{L_U} = 0$, where w_{L_U} denotes the weight or information contribution of the network). Simultaneourly, the information transferred from the rows of $\boldsymbol{R}$ is a maximum, $w_R = 1$. This scenario also leads to a minimum for the total node degree, d_{min}.

On the other hand, if γ_1 is high enough we expect a hypothetical maximum degree, d_{max}, where most of the edges from the network are also observed within the clusters. Thus, the information transferred from $\boldsymbol{L_u}$ is a maximum ($w_{L_U} = 1$) and the information transferred from the rows of $\boldsymbol{R}$ is null, $w_R = 0$. At that level of penalization, d_{max} is approximately the total node degree in the network.

Now we use the observed node degree at γ_1 to weight the information transferred into $\boldsymbol{U}$:

$$w_{L_U} = \frac{d - d_{min}}{d_{max} - d_{min}} \tag{S23}$$

We set γ_1 to assure that the network contribute with some weight (grid search $w_{L_U} = 0.1, 0.3, 0.5$) (Fig S5B). Then, in the final results, we observe the set of prioritised loci and select the weight that yields the highest recovery of reported loci-trait (S6).

S9.2 Information transferred into $\boldsymbol{V}$

As γ_2 increases cNMTF tunes the cluster membership to make it more similar to the phenotype labels. Under population structures, γ_3 will make the final solution of $\boldsymbol{V}$ less similar to population labels (Fig 3B and C). Hence, we have three partitions of m subjects:

- Cluster membership: $\boldsymbol{z}_v = [z_{v,1}, \dots, z_{v,m}]; z_{v,i} \in \{\text{cluster } 1, \dots, \text{cluster } k_2\}$
- Phenotype labels: $\boldsymbol{z}_o = [z_{o,1}, \dots, z_{o,m}]; z_{o,i} \in \{\text{case}, \text{control}\}$
- Population labels: $\boldsymbol{z}_a = [z_{a,1}, \dots, z_{a,m}]; z_{l,i} \in \{\text{population } 1, \dots, \text{population } n\}$

Any change in $\boldsymbol{V}$ as a result of γ_2 and γ_2 is tracked with the normalized Mutual Information (NMI), which quantifies the overlap between partitions (equation S24) [8].

$$NMI(A,B) = \frac{-2\sum_{i=1}^{D}\sum_{j=1}^{E} M_{ij} log(mM_{ij}/M_{i.}M_{.j})}{\sum_{i=1}^{D} M_{i.} log(M_{i.}/m) + \sum_{j=1}^{S} M_{.j} log(M_{.j}/m)} \tag{S24}$$

Where A and B are two partitions of m individuals (*e.g*, z_v and z_o). M is a confusion matrix of size $D \times E$ denoting the number of individuals that partitions A and B share. If A and B do not share individuals among their partitions then $NMI = 0$. If A and B are the same partition, then $NMI = 1$ [9].

We track the similarity cluster-phenotype $NMI(\boldsymbol{z}_v, \boldsymbol{z}_o)$ and the similarity cluster-population $NMI(\boldsymbol{z}_v, \boldsymbol{z}_a)$ when γ_2 and γ_3 increase. Consequently, the weight of the information transferred into the final results (w_{Vo},w_A), is a function of the NMI:

$$w_{Vo} = \frac{NMI(\boldsymbol{z}_v, \boldsymbol{z}_o) - 1}{1 - NMI(\boldsymbol{z}_v, \boldsymbol{z}_o)_{min}} \tag{S25}$$

$$w_A = \frac{NMI(\boldsymbol{z}_v, \boldsymbol{z}_a)_{max} - NMI(\boldsymbol{z}_v, \boldsymbol{z}_a)}{NMI(\boldsymbol{z}_v, \boldsymbol{z}_a)_{max}} \tag{S26}$$

Where $NMI(\boldsymbol{z}_v, \boldsymbol{z}_o)_{min}$ and $NMI(\boldsymbol{z}_v, \boldsymbol{z}_a)_{max}$ are the NMI observed when $\gamma_2 = 0$ and $\gamma_3 = 0$ respectively. Also note that $NMI(\boldsymbol{z}_v, \boldsymbol{z}_o)_{max} = 1$ and $NMI(\boldsymbol{z}_v, \boldsymbol{z}_a)_{min} = 0$.

In Fig 3B and C, when both parameters are high enough the contribution of $\boldsymbol{V_o}$ and $\boldsymbol{A}$ is maximum, so $w_{V_o} = 1$ and $w_A = 1$. This also means that the information transferred from the columns of $\boldsymbol{R}$ is null, $w_R = 0$. Here we set γ_2 to achieve maximum separation of cases-controls, while ignoring clustering patterns from $\boldsymbol{R}$ ($w_R = 0, w_{V_o} = 1$). We also set γ_3 to be maximum while reducing the ancestry information transferred from $\boldsymbol{R}$ ($w_R = 0, w_A = 1$).

S10 Correcting for population structures in LRMs

Logistic Regression Models (LRMs) estimate the probability of having the phenotype (p) in agreement with the individuals' genotypes, while controlling the covariates' effect. The SNV genotypes (X_s) explain the odds of the dependent case/control phenotype (Y_i) by means of a logit function:

$$Logit(P(Y_i = 1)) = \beta_0 + \sum_{j=1}^{J} \beta_j \psi_{i,j} + \beta_{J+1} X_{s,i} \tag{S27}$$

In equation S27, the first J PCs are added to correct for population structures, along with the genotypes, X_s, observed at loci s. Regression parameters, $\boldsymbol{\beta}$, accompany the main variables to evaluate the association between the SNV and phenotype Y.

Confounding for population stratification is evaluated with the genomic control factor λ_{GC}. Once computed the n associations tests between variants and phenotype, the factor λ_{GC} contrasts the median of association statistics (*e.g*, $\chi^2_{(1),i}$, $i = 1 \dots n$) with the theoretical median under the null distribution [10].

$$\lambda_{GC} = \frac{median(\chi^2_{(1),1}, \chi^2_{(1),2}, \chi^2_{(1),3} \cdots \chi^2_{(1),n})}{0.456} \tag{S28}$$

If $\lambda_{GC} > 1$ the statistics are inflated due to confounding, so the statistics can be corrected by $\chi^2_{(1),i}/\lambda_{GC}$ [11].

S11 Performance of cNMTF under population structures

We explored the genetic distances between subjects by conducting multidimensional scaling (MDS) in the genotyping data of each cohort (Fig S18). Overall, the distribution of individuals in all MDS plots matched their population origin (*i.e*, geographical region, ancestry or race). For instance, the structures in the Finnish cohort correspond to local regions in that country and was consistent with a previous analysis [4]. For the white Americans, we inferred their closest ancestry by merging their genotypes with the 1,000 Genomes Project data, conducted k-means clustering on the reduced dimensional space and analysed the cluster composition (Supplementary Table S7). All the white Americans were clustered with subjects of north and south Europe. We also generated a synthetic example of high genetic drifting by merging the white and African Americans from eMERGE cohort. This mixed cohort was only used to evaluate the performance of the method under extreme population structures.

We evaluated the robustness of our method to identify and correct for population structures by comparing the results of corrected and uncorrected versions of cNMTF. In cNMTF we add/remove the correction in the objective function with the weighting parameter γ_3 (Corrected: $\gamma_3 > 0$, Uncorrected: $\gamma_3 = 0$) Any confounding effect due to population structures was detected by the Normalised Mutual Information (**NMI**) similarity. Thus, we compared the overlapping between the clusters of individuals, their population origin and phenotype.

For Finnish and white American individuals our results show 100% similarity between clusters of subjects and the phenotypes due to the low genetic drifting described above (Fig S18, bar plots). In contrast, for the mixed cohort of white/African Americans, strong population structures diminished the similarity cluster-phenotype. In this mixed cohort we also observed a higher similarity between subjects and population origin. Only when the corrected version of cNMTF was used, we successfully increased the similarity with the phenotype and reduced the number of false positive SNV-trait associations (Figures S19, S21).

S12 Correcting population structures in simulated GWAS data

We generated datasets having different settings or combinations of SNVs associated with the phenotype and the population, as described in section S2. Then, we conducted PCA on each dataset. Depending on the combination of SNVs, we observed clusters of subjects in the PCs space. For ex-

ample, there are not visible clusters where the data do not contain population-associated SNVs (S21, settings 1 and 3). In contrast, there are separated clusters in settings 2, 3 and 4, where the data contain between 0.1% and 0.2% of population-associated SNVs.

To quantify the behaviour of cNMTF on these datasets, we assessed the NMI for the corrected and uncorrected clustering results of cNMTF. With regards of the uncorrected results, if the dataset has population-associated SNVs, the overlap between clusters and population is maximum (settings 2 and 4). If the dataset includes 0.1% SNVs associated with phenotype (settings 3 and 5), we observe a better overlap with the phenotype labels, which means that separation of cases and controls is augmented if the data lack confounders. In the corrected results, the algorithm was able to reduce the overlapping with the population in those datasets having strong structures (settings 2 and 4).

All in all, the corrected algorithm led to better clustering similarity with the phenotype, while reducing the overlapping with the population origin.

References

[1] Price, A. L., Patterson, N. J., Plenge, R. M., Weinblatt, M. E., Shadick, N. A., et al. 2006. Principal components analysis corrects for stratification in genome-wide association studies. *Nature Genetics*, 38(8):904–909.

[2] Liu, L., Zhang, D., Liu, H., and Arendt, C. 2013. Robust methods for population stratification in genome wide association studies. *BMC Bioinformatics*, 14(1):132.

[3] Price, A. L., Zaitlen, N. A., Reich, D., and Patterson, N. 2010. New approaches to population stratification in genome-wide association studies. *Nature reviews. Genetics*, 11(7):459–63.

[4] Sabatti, C., Service, S. K., Hartikainen, A.-L., Pouta, A., Ripatti, S., et al. 2009. Genome-wide association analysis of metabolic traits in a birth cohort from a founder population. *Nature Genetics*, 41(1):35–46.

[5] Zhong, Y., Xuan, P., Wang, X., Zhang, T., Li, J., et al. 2018. A non-negative matrix factorization based method for predicting disease-associated miRNAs in miRNA-disease bilayer network. *Bioinformatics*, 34(2):267–277.

[6] Boutsidis, C. and Gallopoulos, E. 2008. SVD-based initialization: A head start on nonnegative matrix factorization. *Pattern Recognition*, 41(4):1350–1362.

[7] Gligorijevic, V., Malod-Dognin, N., and Przulj, N. 2016. Patient-specific data fusion for cancer stratification and personalised treatment. *Biocomputing*, (January):321–332.

[8] Lancichinetti, A., Fortunato, S., and Kertész, J. 2009. Detecting the overlapping and hierarchical community structure in complex networks. *New Journal of Physics*, 11(3):033015.

[9] Amelio, A. and Pizzuti, C. Is Normalized Mutual Information a Fair Measure for Comparing Community Detection Methods? In *Proceedings of the 2015 IEEE/ACM International Conference on Advances in Social Networks Analysis and Mining 2015 - ASONAM '15*, pages 1584–1585. ACM Press, New York, New York, USA 2015.

[10] Devlin, B. and Roeder, K. 1999. Genomic control for association studies. *Biometrics*, 55(4):997–1004.

[11] Dadd, T., Weale, M. E., and Lewis, C. M. 2009. A critical evaluation of genomic control methods for genetic association studies. *Genetic Epidemiology*, 33(4):290–298.

Table S1: Metabolic traits and confounder variables in the cohorts.

Variable †	Finnish	White American
Number of subjects ‡	3,980	1,831
LDL-C (mg/dL)	112.1 (92.81-135.3)	134 (112.8-155)
HDL-C (mg/dL)	58.39 (49.11-68.83)	48 (40-57)
TG (mg/dL)	86.73 (64.6-124.8)	106 (75-150)
Age (years)	31 (31-31)	56 (49-65)
Sex (Male/Female)	M: 48% (1912); F: 52% (2068)	M: 45% (829); F: 55% (1002)
Oral contraceptive	No: 87% (3471); Yes: 13% (509)	-
Pregnancy status	Yes: 2% (68); No: 98% (3912)	-
Alcohol habits (g/day)	4.2 (1.1-11)	-
Smoking habits	Yes: 37% (1467); No: 63% (2513)	Yes: 33% (608); No: 67% (1223)
BMI at birth (kg/m^2)	13.8 (12.94-14.61)	-
BMI (kg/m^2)	23.95 (21.85-26.61)	27.85 (24.9-31.42)

† Continuous variables with non-normal distribution are shown as median and interquartile range in brackets. Categorical variables are summarised by the percentage and the number of subjects in each category. Smoking habits refers to whether or not the subject has ever smoked.

‡ Number of subjects with complete clinical data, including phenotypes and confounders.

Table S2: Classification of lipoproteins and triglycerides levels by the National Institutes of Health (NIH) ‡

LDL-C (mg/dL)	HDL-C (mg/dL)	Triglycerides (mg/dL)
<100 Optimal	<40 Low	<150 Normal
100-129 Above optimal	>60 High	150-199 Borderline high
130-159 Borderline high		200-499 High
160-189 High		≥500 Very high
>190 Very high		

‡ ATP III Guidelines, NIH Publication. In this work, the red and green cells are the cut-off levels chosen for cases and controls, respectively. https://www.nhlbi.nih.gov/files/docs/guidelines/atglance.pdf

Table S3: Coefficients in a multiple regression model for each trait (trait $\sim$ intercept + confounder variables + residuals)

	Finnish						White Americans					
	LDL-C		HDL-C		TG		LDL-C		HDL-C		TG	
	Beta	p-value	Beta	p-value	Beta	p-value	Beta	p-value	Beta	p-value	Beta	p-value
Intercept	115.6	0	59.6	0	87.4	1E-223	119.1	0E+00	47.01	0E+00	94.9	7E-95
Alcohol	-0.1	5E-03	0.1	3E-18	0.3	3E-07	-	-	-	-	-	-
SOCPG-2	-14.3	1E-33	8.5	6E-72	-26.2	2E-40	2.8	6E-02	10.0	1E-62	-12.0	6E-05
SOCPG-3	-17.0	2E-24	13.7	2E-91	6.4	2E-02	-	-	-	-	-	-
SOCPG-4	-13.3	2E-03	18.6	7E-28	22.4	2E-03	-	-	-	-	-	-
SOCPG-5	7.4	5E-01	17.9	3E-05	80.5	6E-06	-	-	-	-	-	-
BMIB-2	-1.6	1E-01	0.6	1E-01	-6.0	4E-04	-	-	-	-	-	-
BMI-2	5.7	9E-05	-2.8	2E-06	10.4	1E-05	9.2	8E-06	-4.13	3E-07	17.2	3E-05
BMI-3	13.9	5E-21	-6.3	0.000	23.7	3E-22	13.1	3E-10	-6.27	1E-14	38.5	5E-20
BMI-4	19.3	8E-39	-11.0	2E-76	52.6	3E-98	11.8	1E-08	-8.83	1E-26	48.6	4E-30
Smoking	-	-	-2.0	4E-06	4.7	8E-03	-	-	-	-	-	-
Age-2	-	-	-	-	-	-	5.6	7E-03	2.79	6E-04	8.7	4E-02
Age-3	-	-	-	-	-	-	8.9	2E-05	3.10	1E-04	6.8	1E-01
Age-4	-	-	-	-	-	-	7.0	6E-04	1.92	2E-02	10.8	1E-02

A dash "-" is showed if the confounder was not included in the best model. Variable SOCPG summarises the sex, pregnancy status and use of oral contraceptives (SOCPG = 1, Male; SOCPG = 2; Female/No pregnant/No contraceptive; SOCPG = 3, Female/No Pregnant/Contraceptive; SOCPG = 4, Female/Pregnant/No Contraceptive, SOCPG = 5, Female/Pregnant/Contraceptive). Variables BMI and age were categorised by placing the ordered values in four quantiles. BMI at birth (BMIB) was categorised by placing the ordered values in two quantiles.

Table S4: Optimal cNMTF parameters

	Finnish			White American		
**Variable**	**LDL-C**	**HDL-C**	**TG**	**LDL-C**	**HDL-C**	**TG**
Number of clusters of SNVs, k_1	20	20	20	20	20	20
Number of clusters of subjects, k_2	2	2	2	2	2	2
Parameter γ_1	98	936	27	439	44	29
Parameter γ_2	30	60	62	184	255	195
Parameter γ_3	0.25	0.25	0.25	0.25	0.25	0.25
Weight of the SNV-SNV network, w_{L_U}	0.10	0.30	0.10	0.50	0.10	0.10

Table S5: Enriched biological annotations in the prioritised genes.

**Enriched biological annotation** ‡	**Trait**	**Cohort**	p**-value** †
IPR012171:Fatty acid/sphingolipid desaturase	LDL-C	Finnish	4E-03
IPR005804:Fatty acid desaturase, type 1	LDL-C	Finnish	2E-02
IPR012171:Fatty acid/sphingolipid desaturase	TG	Finnish	5E-04
IPR005804:Fatty acid desaturase, type 1	TG	Finnish	2E-03
IPR001199:Cytochrome b5-like heme/steroid binding domain	TG	Finnish	8E-03
GO:0006636 unsaturated fatty acid biosynthetic process	TG	Finnish	2E-02
GO:0043691 reverse cholesterol transport	HDL-C	White American	2E-02
GO:0045471 response to ethanol	LDL-C	White American	1E-02
hsa04976:Bile secretion	LDL-C	White American	3E-02

‡ Significant functional annotations retrieved from DAVID (`https://david.ncifcrf.gov`). Accession numbers: IPR: INTERPRO, GO: Gene Ontology term of biological processes, hsa: KEGG pathway.
† Adjusted by Bonferroni correction.

Table S6: Configuration of synthetic data with population structures.

SNV type	SNV associated with		Number of SNVs per dataset				
	**Population**	**Phenotype**	**Setting 1**	**Setting 2**	**Setting 3**	**Setting 4**	**Setting 5**
**I**	No	No	10,000	9,990	9,990	9,990	9,970
**II**	Yes	No	0	10	0	0	10
**III**	No	Yes	0	0	10	0	10
**IV**	Yes	Yes	0	0	0	10	10
		**Total SNVs**	10,000	10,000	10,000	10,000	10,000

Table S7: Clustering of white Americans (WA) from eMERGE and 1,000 Genomes Project data. Number of individuals per cluster and their reported ancestry.

Cluster	Description	AFR	AMR	CEU/GBR	EAS	FIN	IBS/TSI	SAS	WA
1	North Europe	0	0	125	0	13	24	0	861
2	South Europe	0	0	65	0	6	189	0	549
3	Africa	657	2	0	0	0	0	0	0
4	America I	0	74	0	0	0	0	0	0
5	America II	1	163	0	0	0	0	0	0
6	America III	3	75	0	0	0	0	0	0
7	East Asia	0	0	0	504	0	0	0	0
8	Finland	0	33	0	0	80	1	0	0
9	Southern Asia	0	0	0	0	0	0	489	0

From 1,000 Genomes Project the following populations and subpopulations were analysed: AMR: Americas, SAS: Southern Asians, EAS: East Asians, AFR: Africans, CEU: Utah residents with Northern and Western European ancestry, IBS: Iberian populations in Spain, FIN: Finnish in Finland, GBR: British in England and Scotland, TSI: Toscani in Italy.

In cluster 1, a total of 861 white Americans were clustered mainly with British in England and Scotland, and Utah residents with Northern and Western European ancestry. Cluster 2 consists of 542 white Americans clustered with individuals from Iberian populations in Spain, and Toscani in Italy.

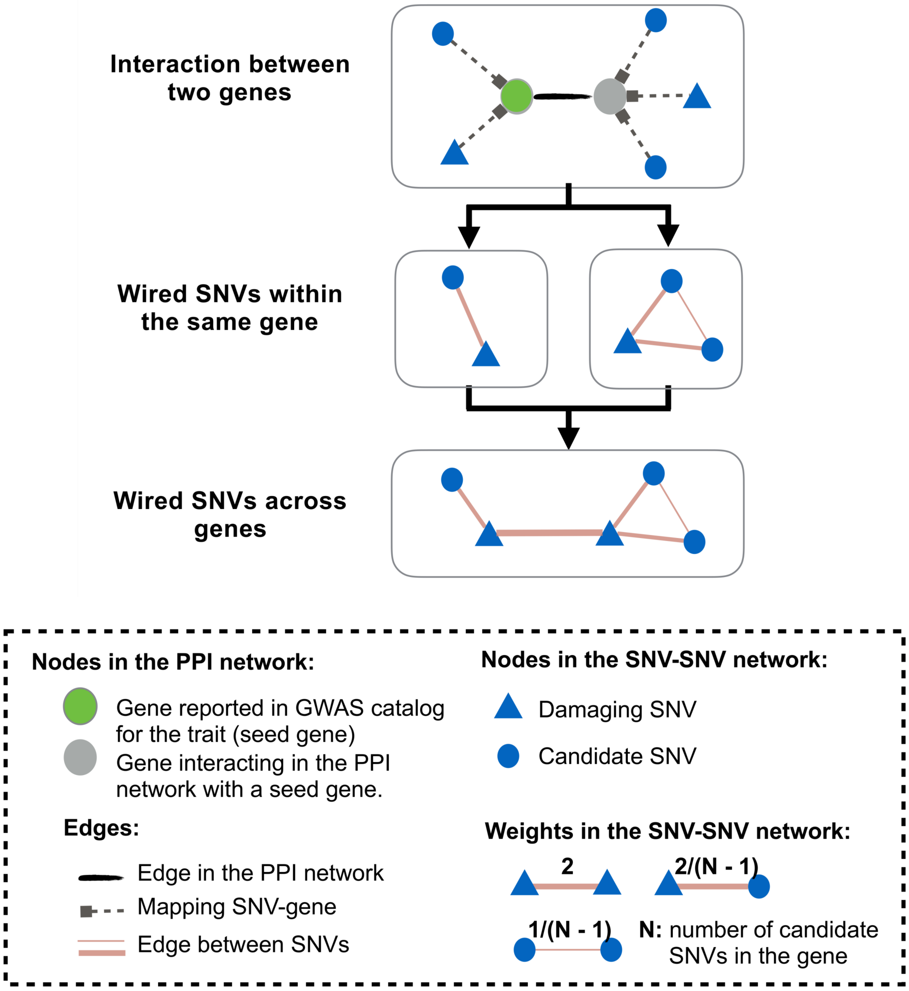

Figure S1: Construction of the SNV-SNV network. The variants under analysis are labelled either as *damaging* or *candidate*. They are connected if they are harboured by the same gene. The edge weight depends on the number of variants within the gene region, so we remove the bias in the node degree when genes harbour thousands of variants. *Damaging* variants are further connected across genes and their edge weight is increased to reflect their effect on the PPI network.

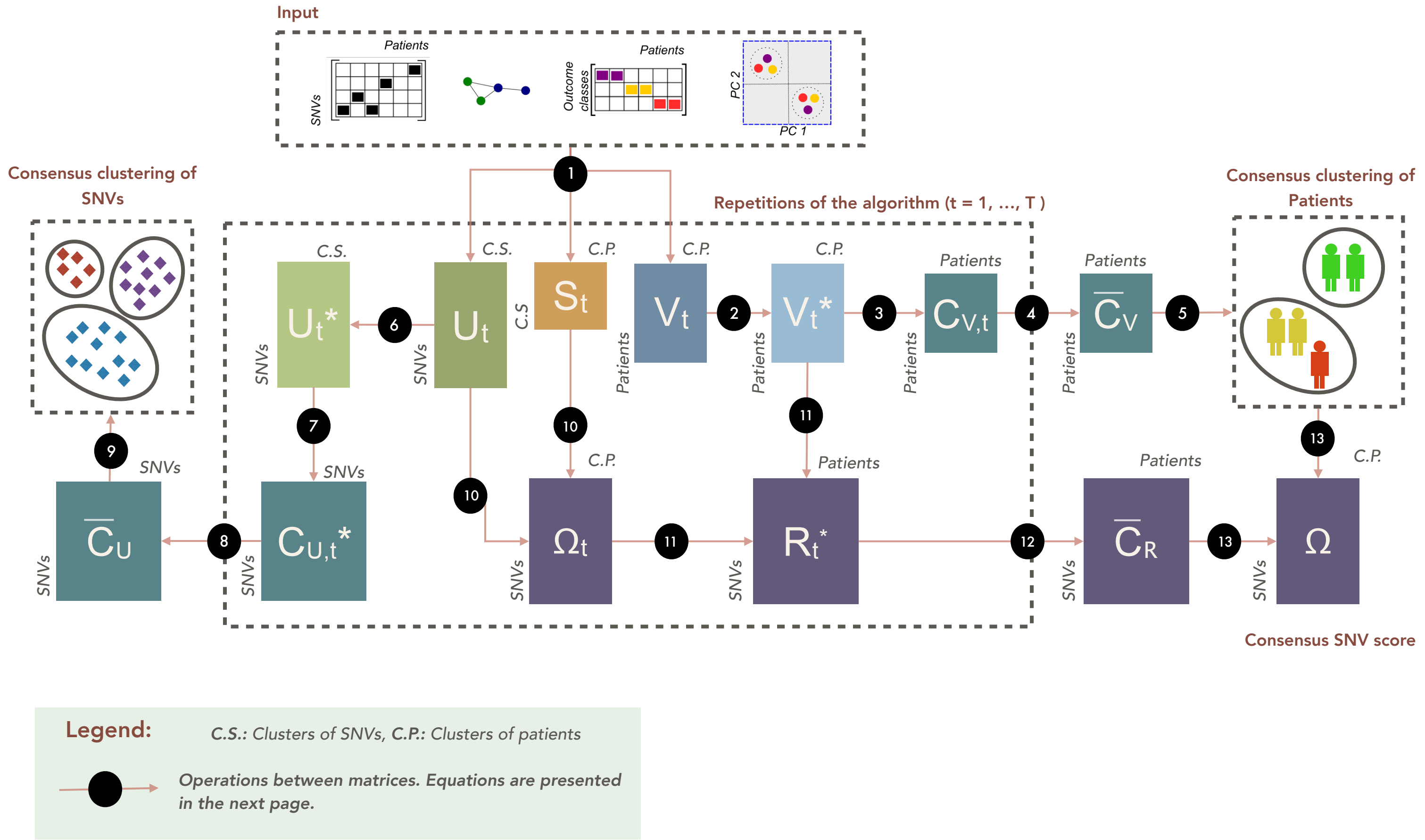

Figure S2: Consensus clustering and consensus SNV score. This scheme shows the mathematical operations to achieve unique results in cNMTF. **(1)** The objective function is minimised T times with different initialisations of $\boldsymbol{U_t}$, $\boldsymbol{S_t}$ and $\boldsymbol{V_t}$ for $t : 1, \ldots, T$. **(2,3)** At each repetition, we find the clustering membership, $\boldsymbol{V_t^*}$, and track subjects (patients) belonging to the same cluster with $C_{V,t}$. **(4,5)** These results are averaged in $\overline{\boldsymbol{C}}_V$, where hierarchical clustering is applied to identify consensus clusters. **(6 - 9)** Same procedures are performed for the clustering of SNVs. Matrix $\boldsymbol{U_t^*}$ captures the clustering membership, while matrices $C_{U,t}$ and $\overline{\boldsymbol{C}}_U$ register variants clustered together. The later consolidates the results across repetitions and is used for hierarchical clustering of SNVs. **(10)** On the other hand, a SNV score matrix $\boldsymbol{\Omega_t}$ is generated by multiplying $\boldsymbol{U_t}$ and $\boldsymbol{S_t}$. **(11)** Because the clusters of subjects might change through repetitions, the SNV scores must be distributed over the individual subjects by multiplying $\boldsymbol{\Omega_t}$ and $\boldsymbol{V_t^*}$. **(12)** $\boldsymbol{R^*}_t$ is calculated and added up through repetitions in a consensus relationship matrix $\overline{\boldsymbol{C}}_R$. **(13)** We obtain consensus SNV scores by assessing the median entries of $\overline{\boldsymbol{C}}_R$ within each cluster of subjects.

Equation labels in figure S2:

$$\min_{U\geq 0, S\geq 0, V\geq 0} J_{cNMTF} = \|\boldsymbol{R} - \boldsymbol{USV}^T\|_F^2 + \gamma_1 \cdot tr(\boldsymbol{U^T L_U U}) + \gamma_2 \cdot \|\boldsymbol{V} - \boldsymbol{V_o}\|_F^2 + \gamma_3 \cdot \mathrm{Tr}\left(\boldsymbol{VV^T HAH}\right) \quad \text{(Label 1)}$$

$$V_t^*[i,j] = \begin{cases} 1 \text{ if } V_t[i,j] = \max\left(V_t[\,j]\right) \\ 0 \text{ otherwise} \end{cases} \quad \text{(Label 2)}$$

$$C_{V,t}[i,l] = \begin{cases} 1 \text{ if } V_t^*[i,] = V_t^*[l,] \\ 0 \text{ otherwise} \end{cases} \quad \text{(Label 3)}$$

$$\bar{C}_V[i,l] = \frac{1}{T}\sum_{t=1}^{T} C_{V,t}[i,l] \quad \text{(Label 4)}$$

Hierarchical clustering (Label 5)

$$U_t^*[i,j] = \begin{cases} 1 \text{ if } U_t[i,j] = \max\left(U_t[,j]\right) \\ 0 \text{ otherwise} \end{cases} \quad \text{(Label 6)}$$

$$C_{U,t}[i,l] = \begin{cases} 1 \text{ if } U_t^*[i,] = U_t^*[l,] \\ 0 \text{ otherwise} \end{cases} \quad \text{(Label 7)}$$

$$\bar{C}_U[i,l] = \frac{1}{T}\sum_{t=1}^{T} C_{U,t}[i,l] \quad \text{(Label 8)}$$

Hierarchical clustering (Label 9)

$$\varOmega_t = U_t S_t \tag{Label 10}$$

$$R_t^* = \varOmega_t V_t^* \tag{Label 11}$$

$$\bar{C}_{R^*} = \frac{1}{T}\sum_{t=1}^{T} R_t^* \tag{Label 12}$$

$$\begin{gathered}\varOmega[i,j] = median(\bar{C}_{R^*}[i,k]);\\ \text{Subject } k \in \{\text{cluster } j\}\end{gathered} \tag{Label 13}$$

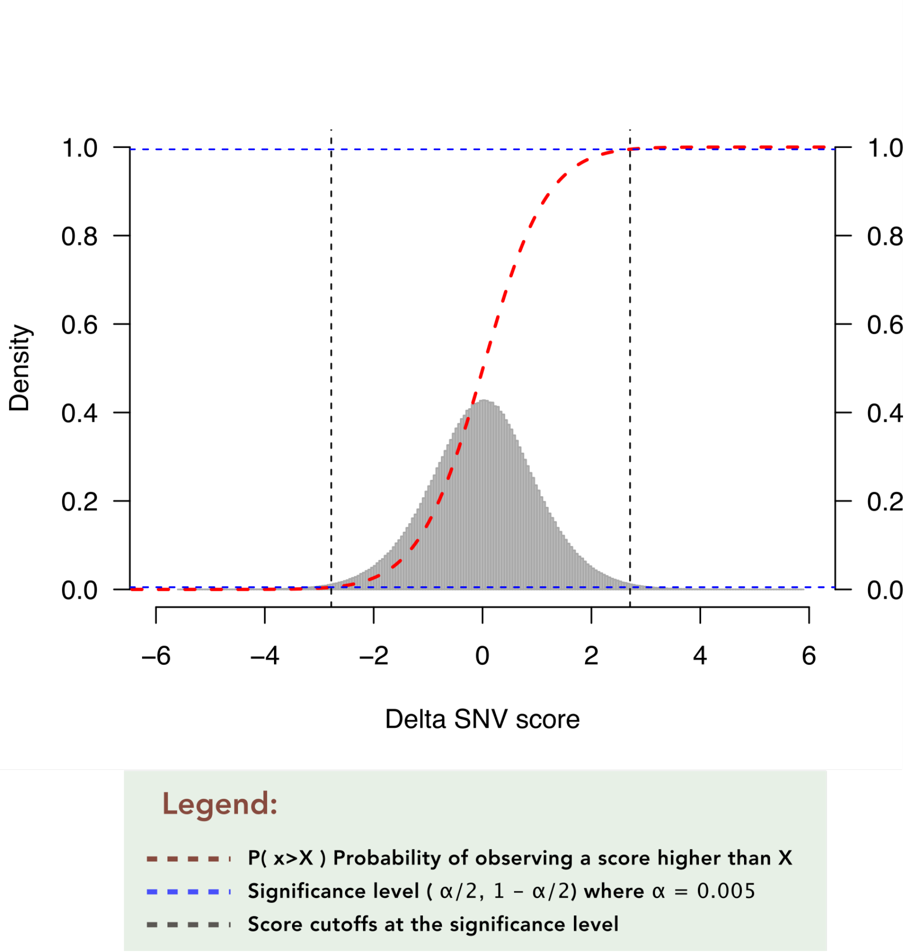

Figure S3: Distribution of the delta SNV score for LDL-C in the Finnish cohort. Dashed vertical lines represent the cut-off points (-2.78, 2.71) to prioritise significantly associated variants in both tails. The cut-off points were defined at significance levels ($\frac{\alpha}{2}, 1 - \frac{\alpha}{2}$) , where $\alpha = 0.01$.

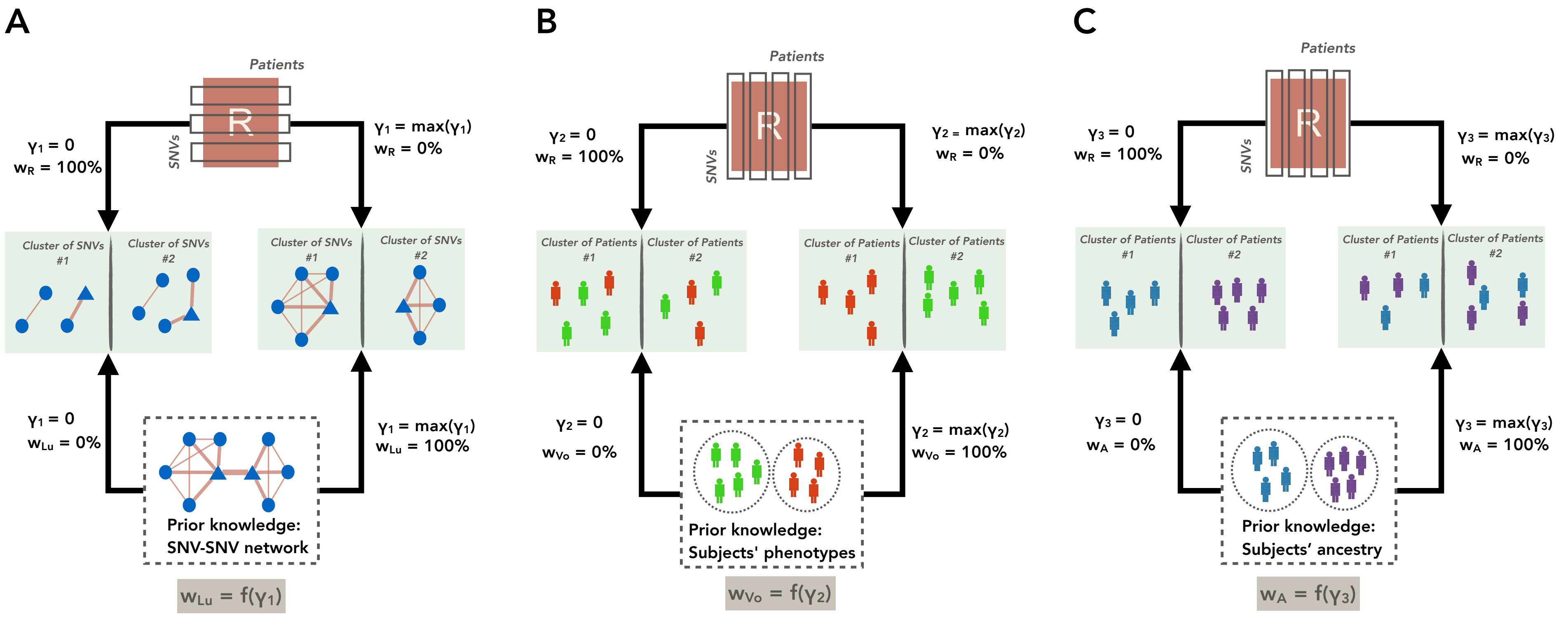

Figure S4: Weighting the information contribution of the genotyping data (w_R) and the prior knowledge (w_{L_u}, w_{V_o}, w_A) in the final results. **(A)** Parameter γ_1 weights the information from the SNV-SNV network that is transferred into the clusters. When $\gamma_1 = 0$ the clusters gather together SNVs with similar row-vectors in $\boldsymbol{R}$. In contrast, if γ_1 is large enough, the observed clusters correspond to highly connected communities from the network and the information from $\boldsymbol{R}$ is ignored. We set γ_1 to ensure that the network contributes to some extent to the final SNV clusters, and selected the w_{L_u} that maximizes the number of known loci-trait associations in the results. **(B)** Parameter γ_2 weights the information from the phenotype $\boldsymbol{V_o}$ that is transferred into the clusters of subjects. We aim at identifying SNVs that better segregate cases and controls, so we set γ_2 to achieve maximum separation of cases-controls, while reducing the clustering patterns from $\boldsymbol{R}$ ($w_R = 0, w_{V_o} = 1$). **(C)**, Parameter γ_3 weights the information from the subjects' ancestry. The goal with this parameter is to minimise the effect of population structures embedded in $\boldsymbol{R}$, which leads to clusters enriched in subjects of the same origin. We set γ_3 to be maximum while reducing the ancestry information transferred from $\boldsymbol{R}$ ($w_R = 0, w_A = 1$).

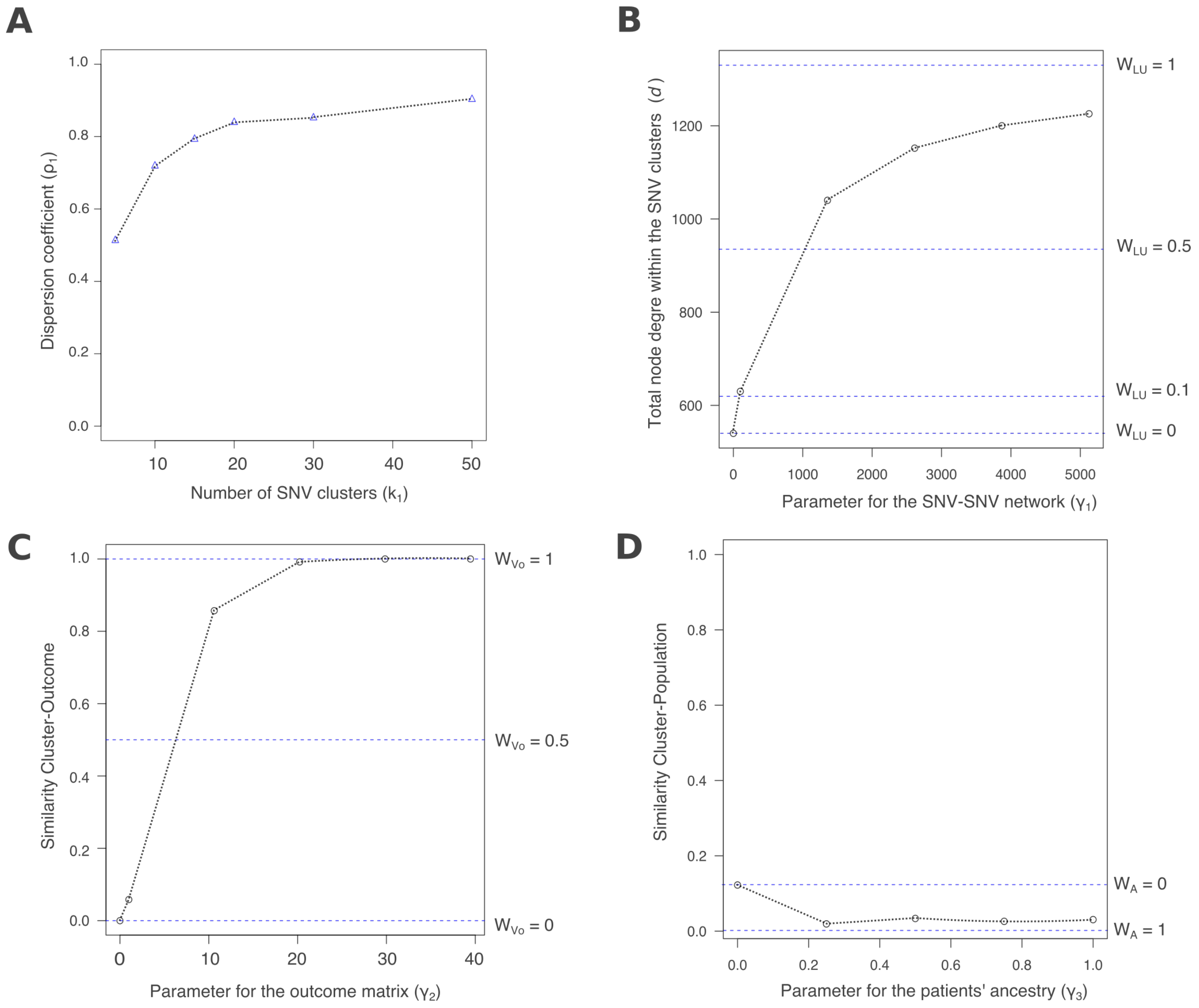

Figure S5: Selection of optimal parameters. **(A)** The number of SNV clusters is selected by tracking the dispersion coefficient, ρ_{k_1}. This coefficient reaches a plateau around $k_1 = 20$ clusters, which is the optimum value selected (LDL-C, Finnish cohort). **(B)** Change in the total node degree at different values of γ_1. When γ_1 increases, more information is contributed by the network and the total node degree rises. For $w_{L_U} = 0.1$, an optimal $\gamma_1 = 98$ is found (see equation S22). (LDL-C, Finnish cohort) **(C)** Similarity between clusters of subjects and their phenotype when γ_2 increases. We selected $\gamma_2 = 30$ to maximise the separation of case-control individuals ($w_{V_o} = 1$) (LDL-C, Finnish cohort). **(D)** Similarity between clusters of subjects and their population origin. At $\gamma_3 = 0.25$ this similarity is minimised ($w_A \sim 1$) (LDL-C, Mixed cohort of white and African Americans)

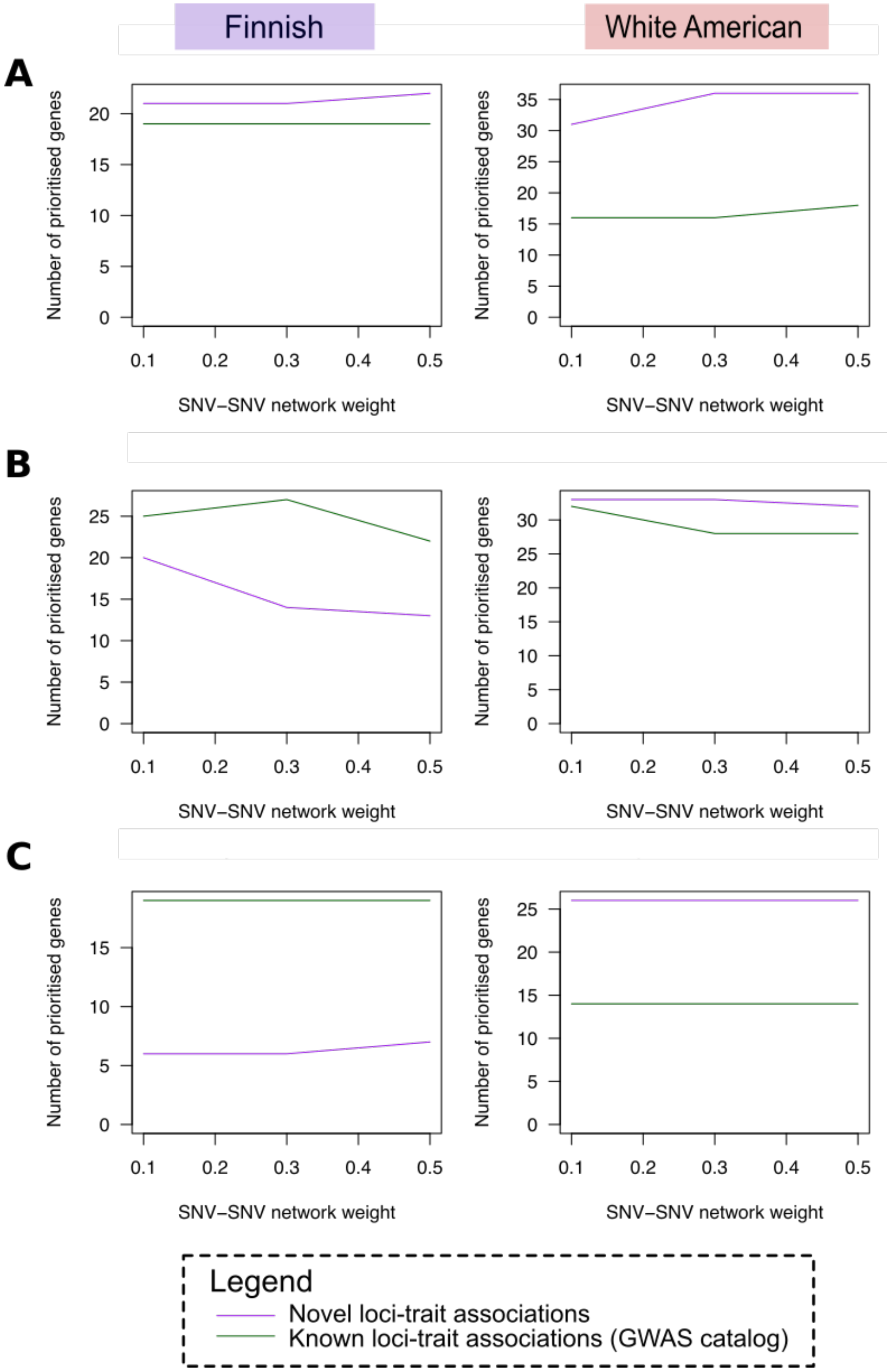

Figure S6: Weighting the network contribution in the final results. Number of prioritised genes by trait at different SNV-SNV network weights ($w_{L_U} = 0.1, 0.3, 0.5$). **(A)**. LDL-C, **(B)**. HDL-C, **(C)**. TG

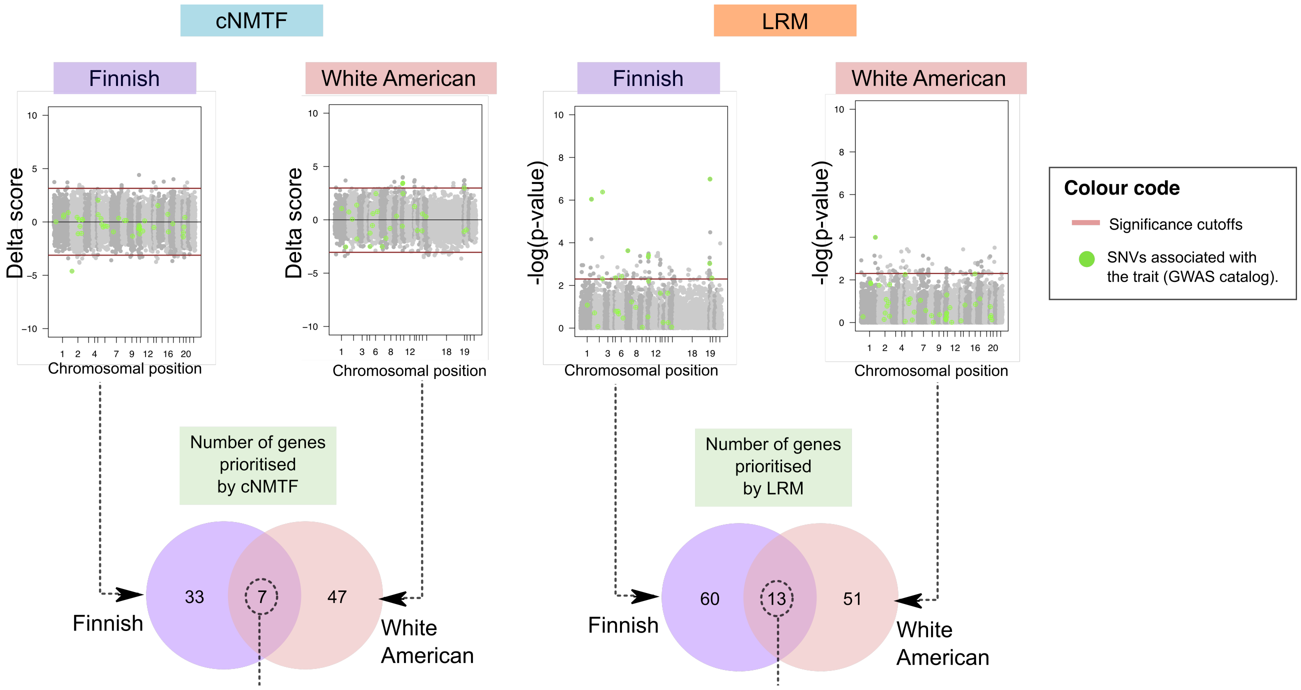

Figure S7: Dispersion of delta scores for LDL-C Following a Manhattan plot layout, variants are ordered according to their chromosomal location. Known associations are highlighted green if reported by GWAS catalog. Variants beyond the significance cut-offs are prioritised by both methods, cNMTF and LRM, and their genes are prioritised for further analysis.

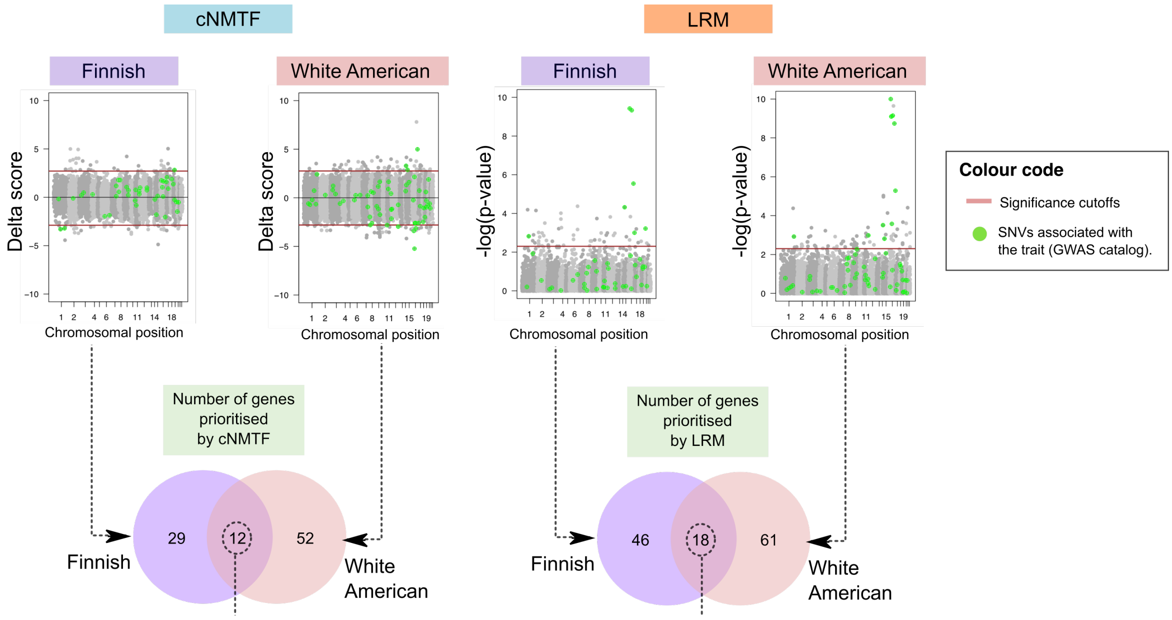

Figure S8: Prioritised genes for HDL-C. Following a Manhattan plot layout, variants are ordered according to their chromosomal location. Known associations are highlighted green if reported by GWAS catalog. Variants beyond the significance cut-offs are prioritised by both methods, cNMTF and LRM, and their genes are prioritised for further analysis. The Venn diagrams highlight those genes prioritised in both cohorts.

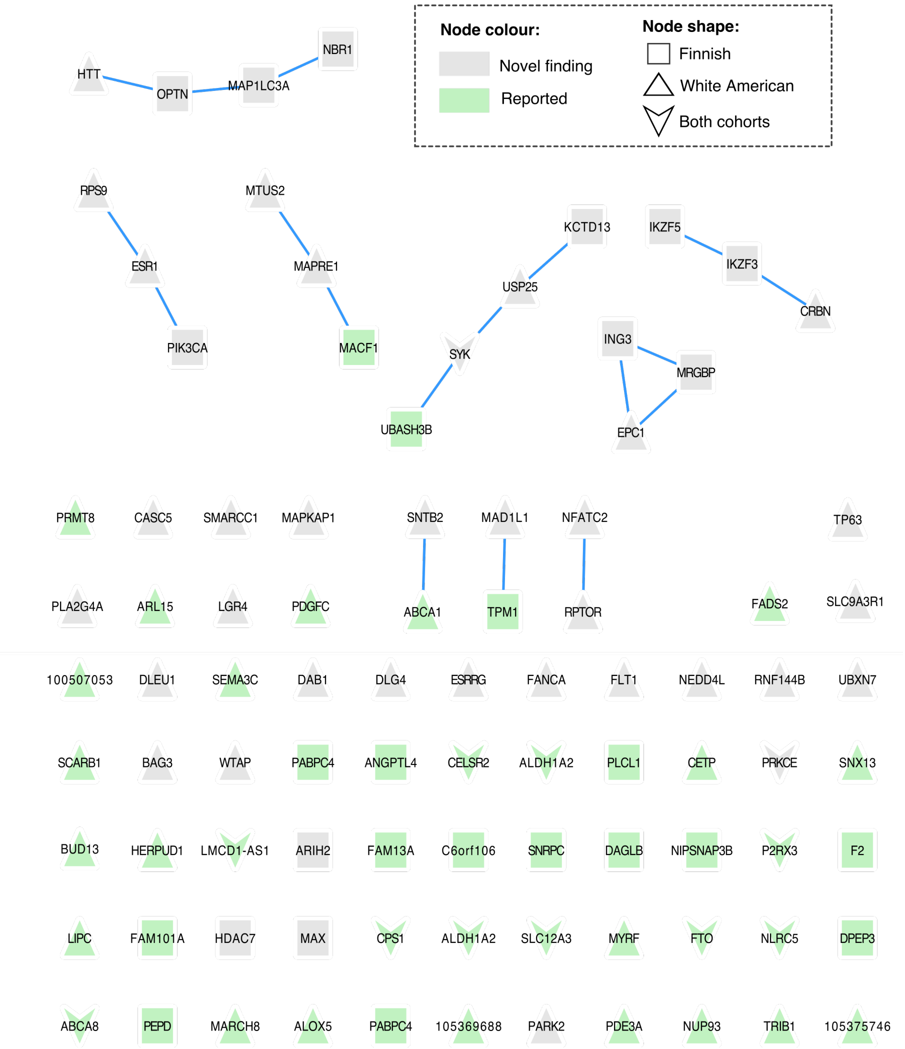

Figure S9: Prioritised protein protein interactions in HDL-C. This PPIN shows only interactions between prioritised genes by cNMTF. Grey nodes represent novel genes either not reported or not significant in GWAS catalog ($p < 10^{-8}$)

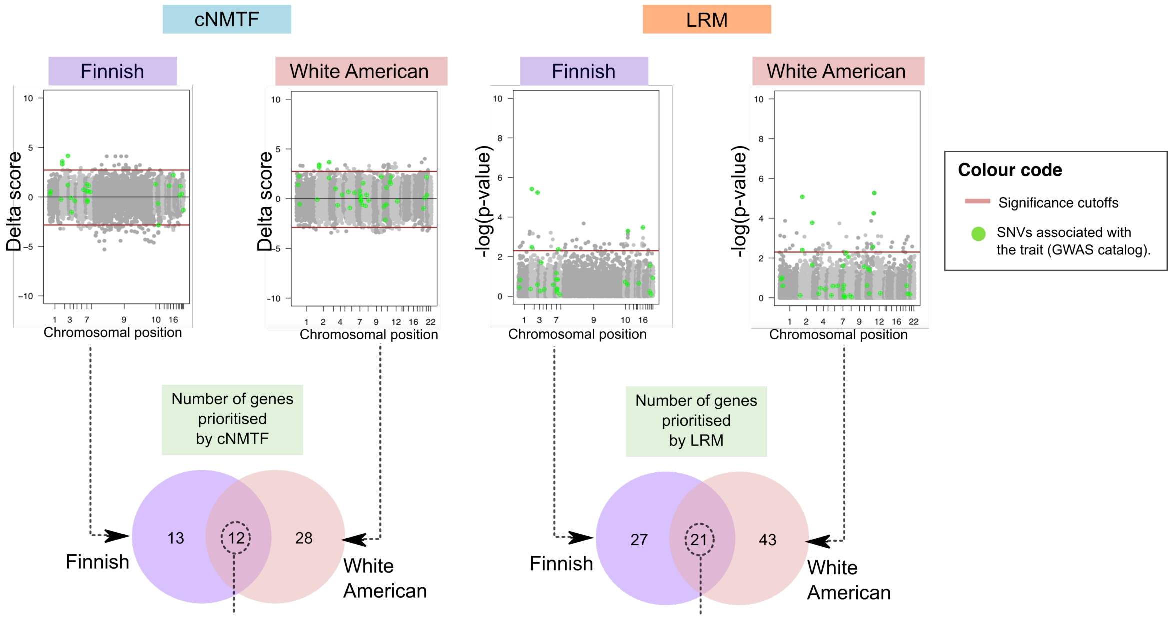

Figure S10: Prioritised genes in TG. Following a Manhattan plot layout, variants are ordered according to their chromosomal location. Known associations are highlighted green if reported by GWAS catalog. Variants beyond the significance cut-offs are prioritised by both methods, cNMTF and LRM, and their genes are prioritised for further analysis. The Venn diagrams highlight those genes prioritised in both cohorts.

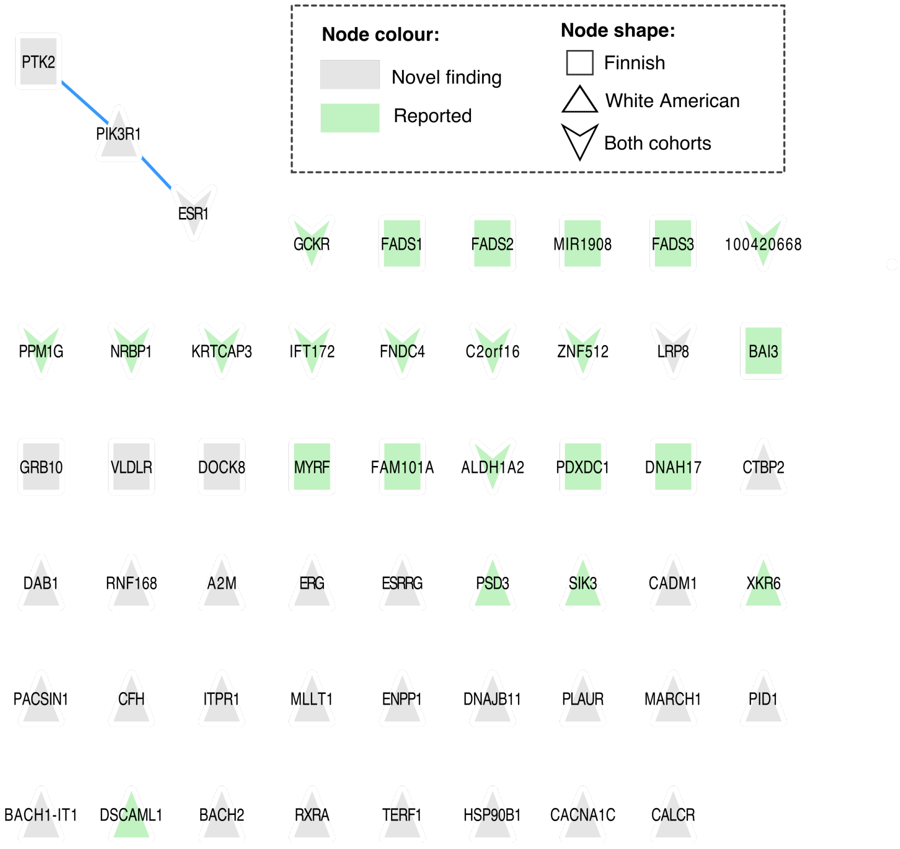

Figure S11: Prioritised protein protein interactions in TG. This PPIN shows only interactions between prioritised genes by cNMTF. Grey nodes represent novel genes either not reported or not significant in GWAS catalog ($p < 10^{-8}$)

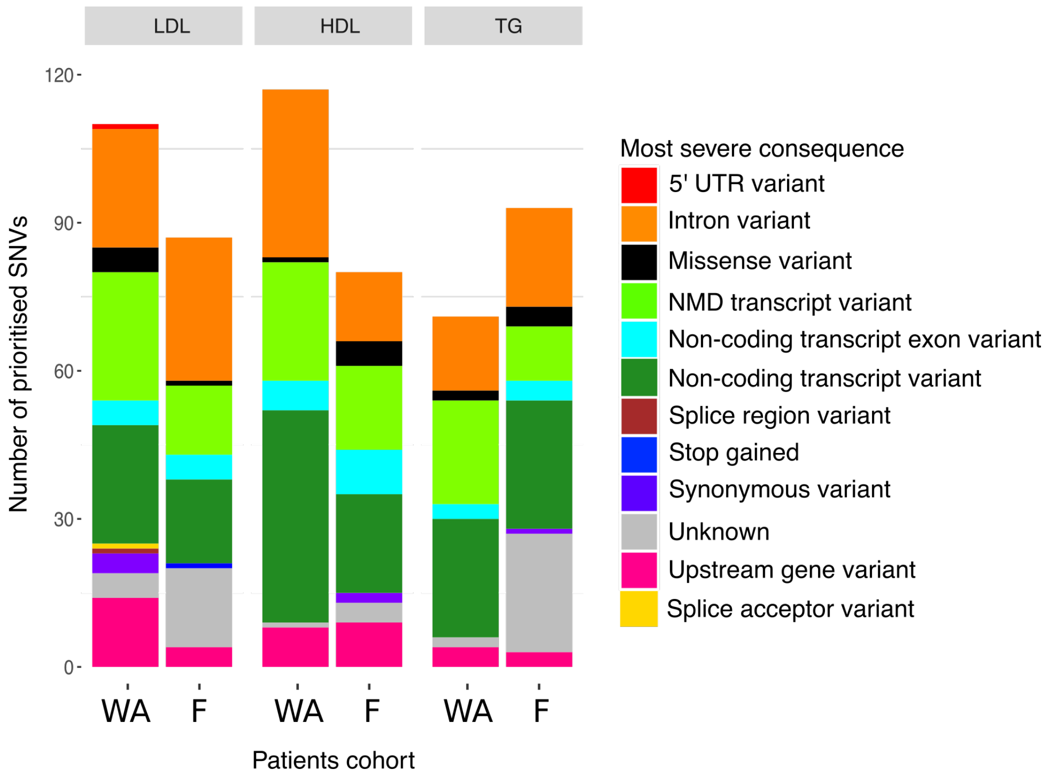

Figure S12: Impact of prioritised variants in the canonical transcripts. The most severe consequence between each prioritised variant and their counterparts in high linkage-disequilibrium was queried from ENSEMBLE. Subjects cohort: Finnish **(F)** and white American **(WA)**

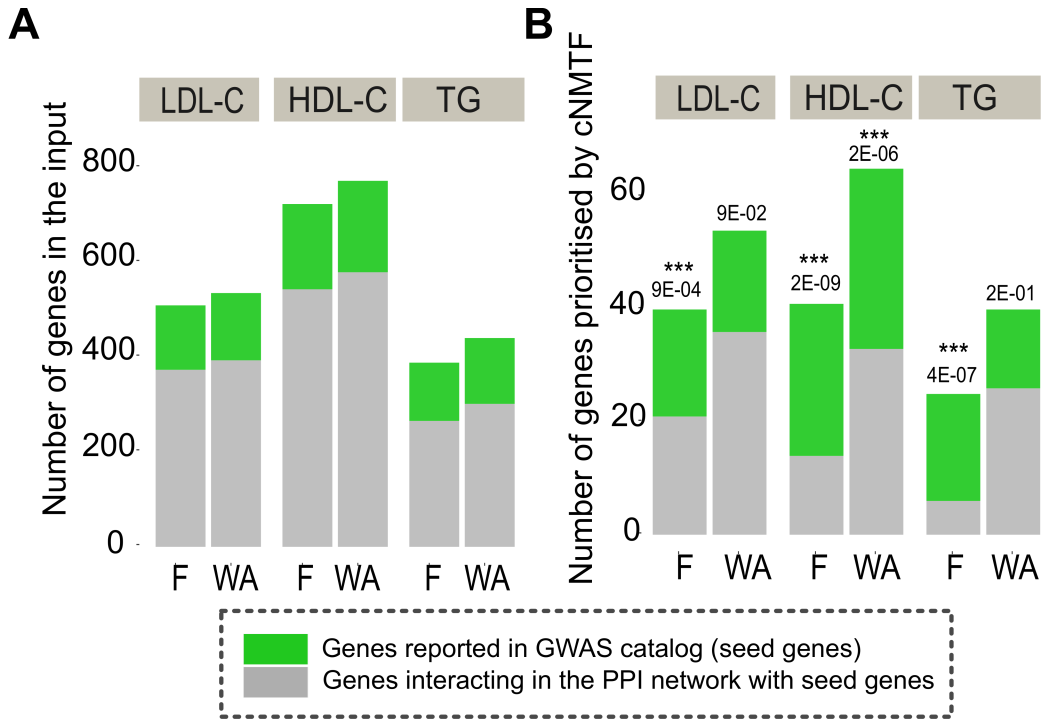

Figure S13: . Genes in the input and results of cNMTF. **(A)** The genes in the input are those harbouring known SNV-trait associations (seed genes), plus their interacting partners in the first neighbourhood of the PPI network. **(B)** We tested the enrichment of seed genes in the results given the input. p-values for the hypergeometric test are listed over the bars (significance code *** $p < 0.001$). F: Finnish, WA: White Americans

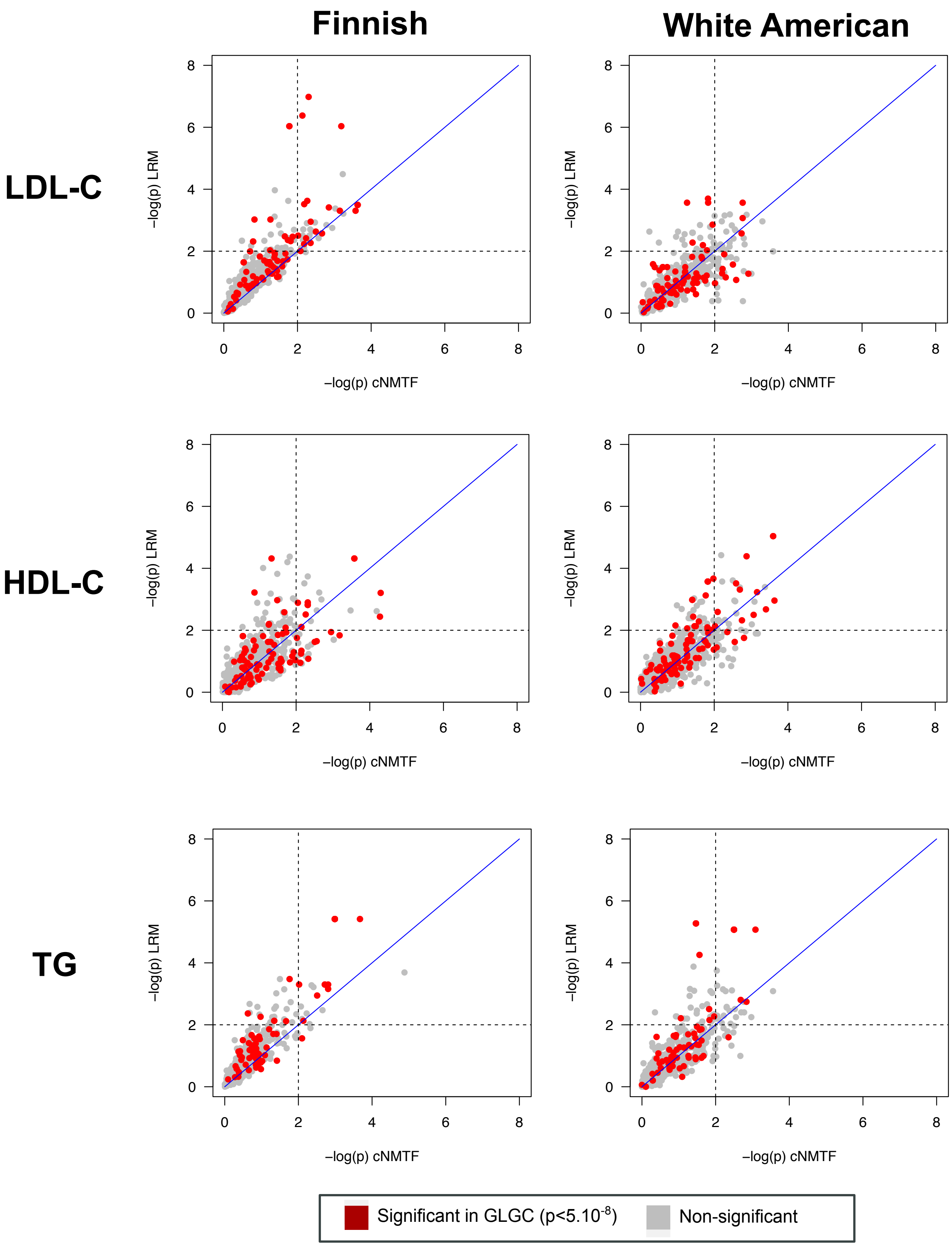

Figure S14: Dispersion of prioritised genes in cNMTF vs LRM. Genes are plotted according to the lowest SNV p-value in each method. Red dots highlight significant genes in the Global Lipids Genetics Consortium.

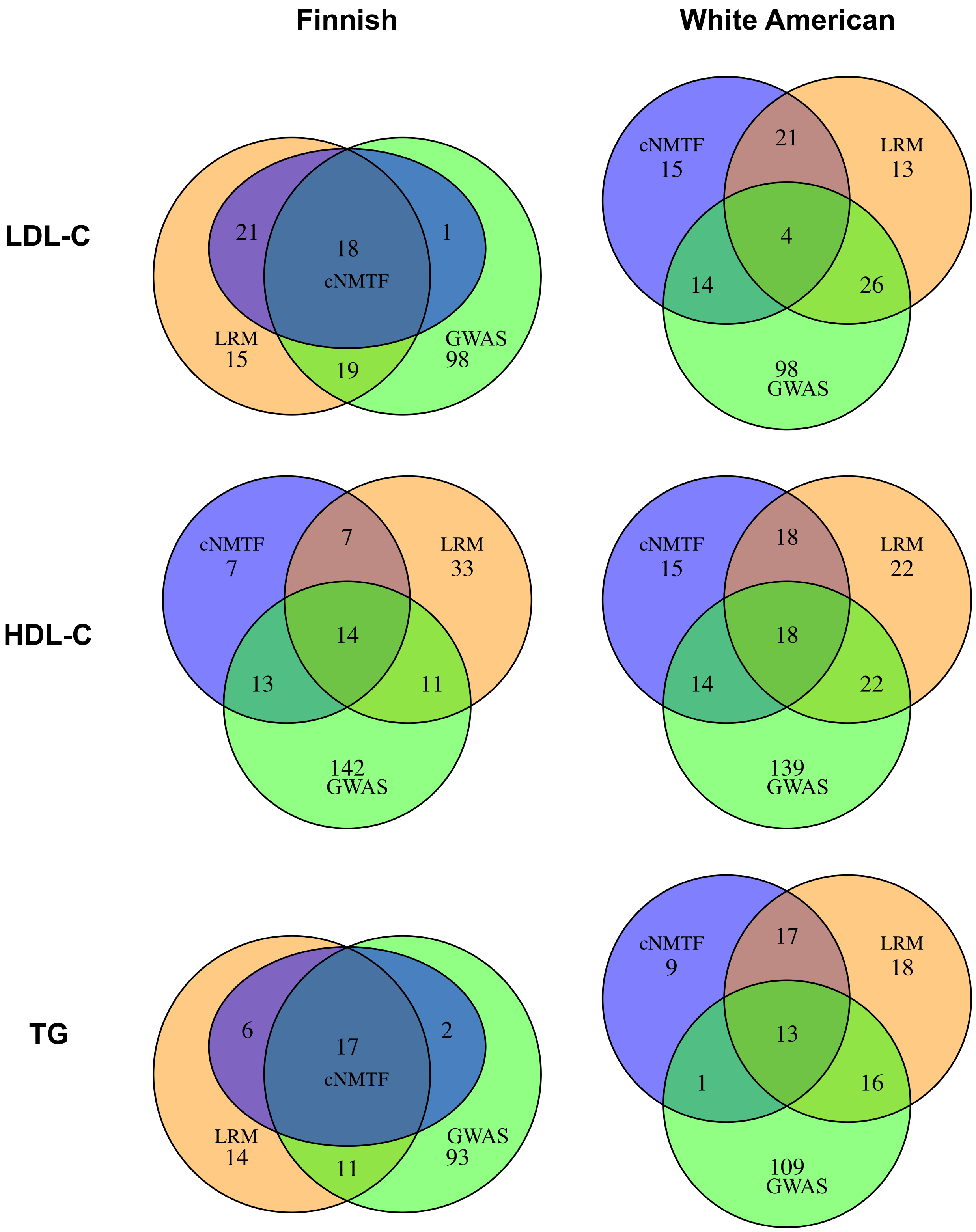

Figure S15: Prioritising lipid-associated genes from GWAS catalog. Genes reported for each trait in GWAS catalog are observed in the results of cNMTF and LRM. (Finnish (F), white American (WA))

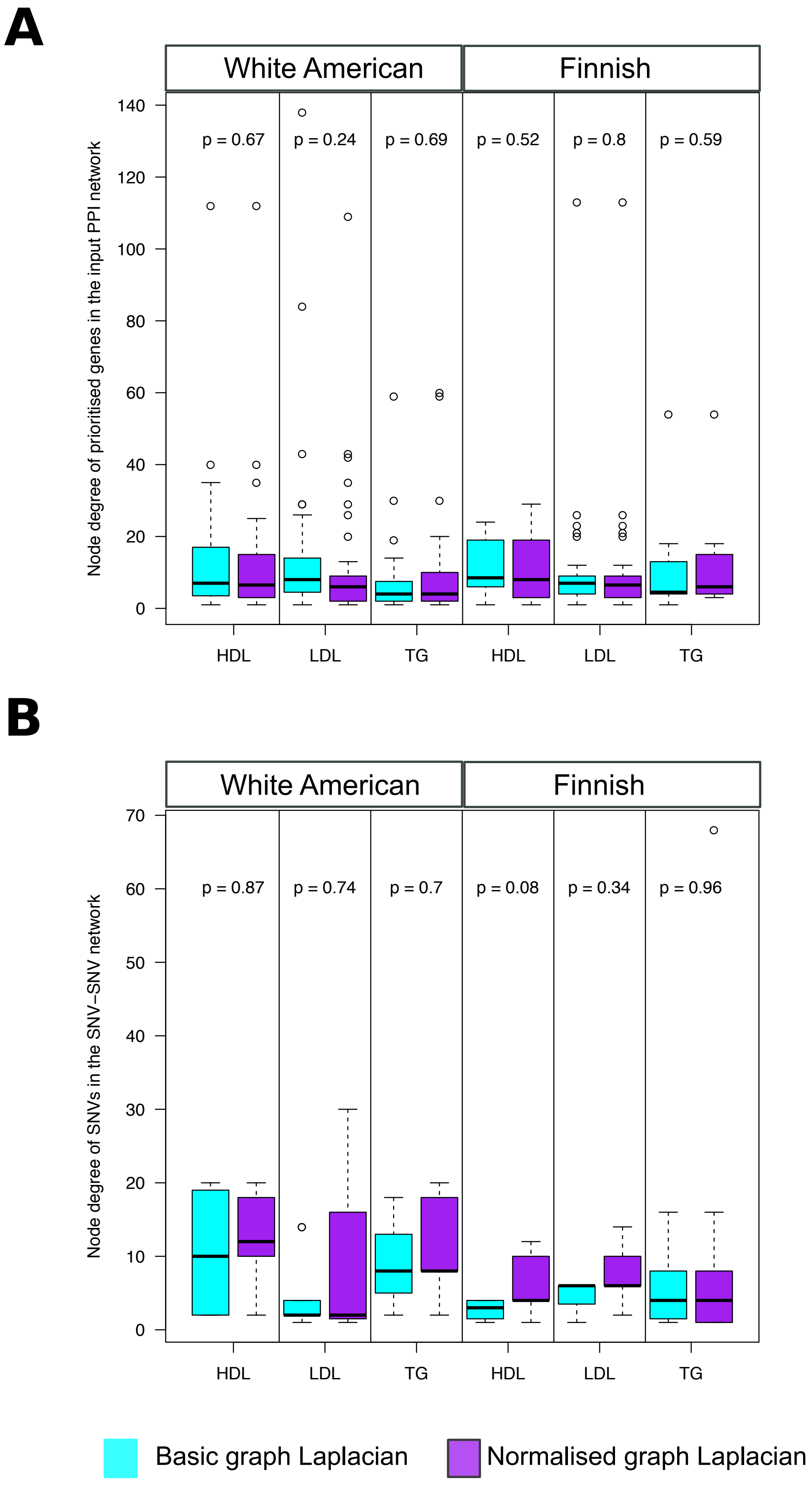

Figure S16: Comparison of basic graph Laplacian and normalised graph Laplacian results. (A) Node degree of prioritised genes in the input PPI network. **(A)** Node degree of prioritised SNVs in the SNV-SNV network as constructed in Figure S1.

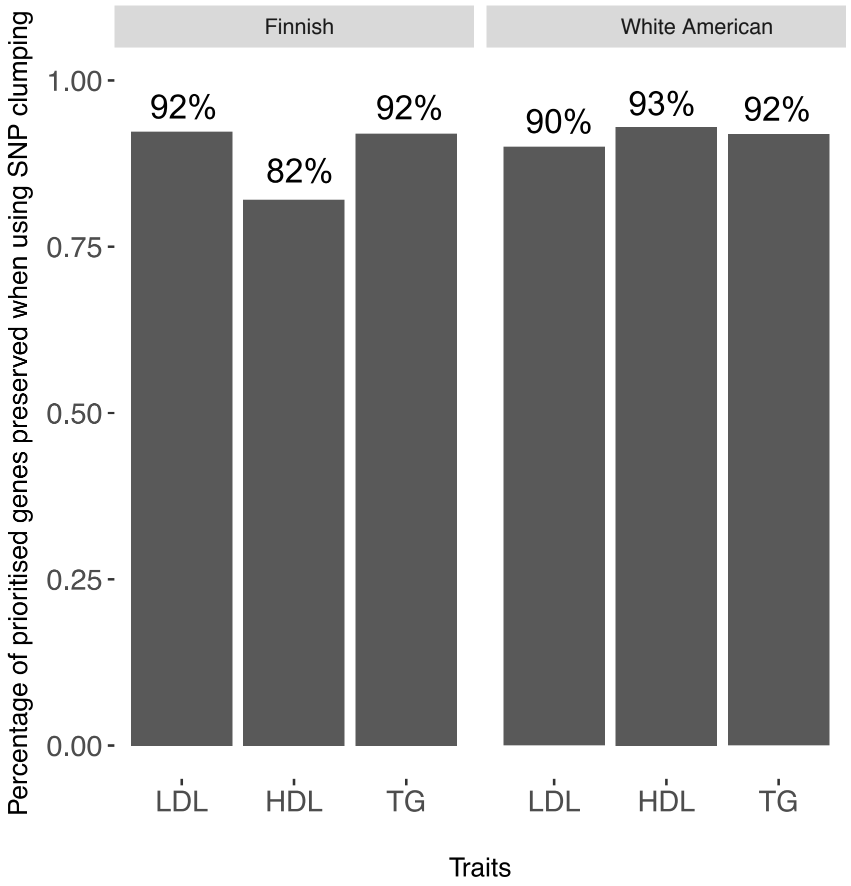

Figure S17: Changes in gene prioritisation when clumping SNVs in high LD. The set of prioritised genes is compared between settings of cNMTF (No clumping Vs Clumping SNVs), and we report the percentage of genes preserved: (number of prioritised genes in the intersection / number of prioritised genes when clumping)

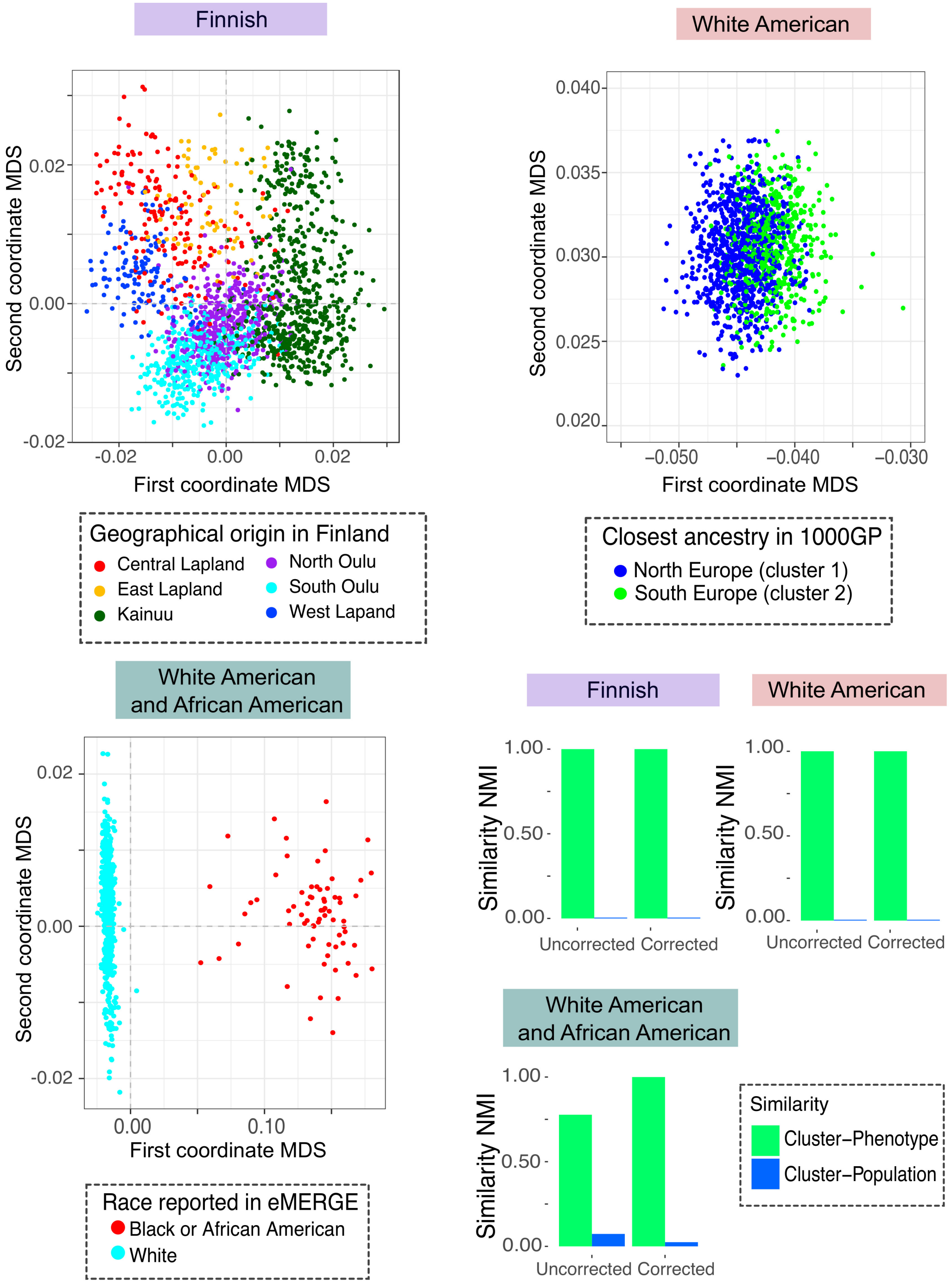

Figure S18: Evaluating the confounding effect of population structures in the clustering results. Multidimensional scaling is conducted on a sample of 15,000 SNVs to visualise population structures. Finnish: The structures match geographical areas in Finland. White American: this cohort was merged with 1000 Genomes Project data and the individuals matched the European ancestry. White American and African American: This is a synthetic example of strong population structures embedded in the data. Both groups of individuals come from the eMERGE network. **Bar plots:** The corrected and uncorrected forms of the algorithms are compared for LDL in both cohorts. The presence of population structures is tracked with the similarity cluster-phenotype and cluster-population. Only the merged cohort (white and African Americans) shows strong confounding effects

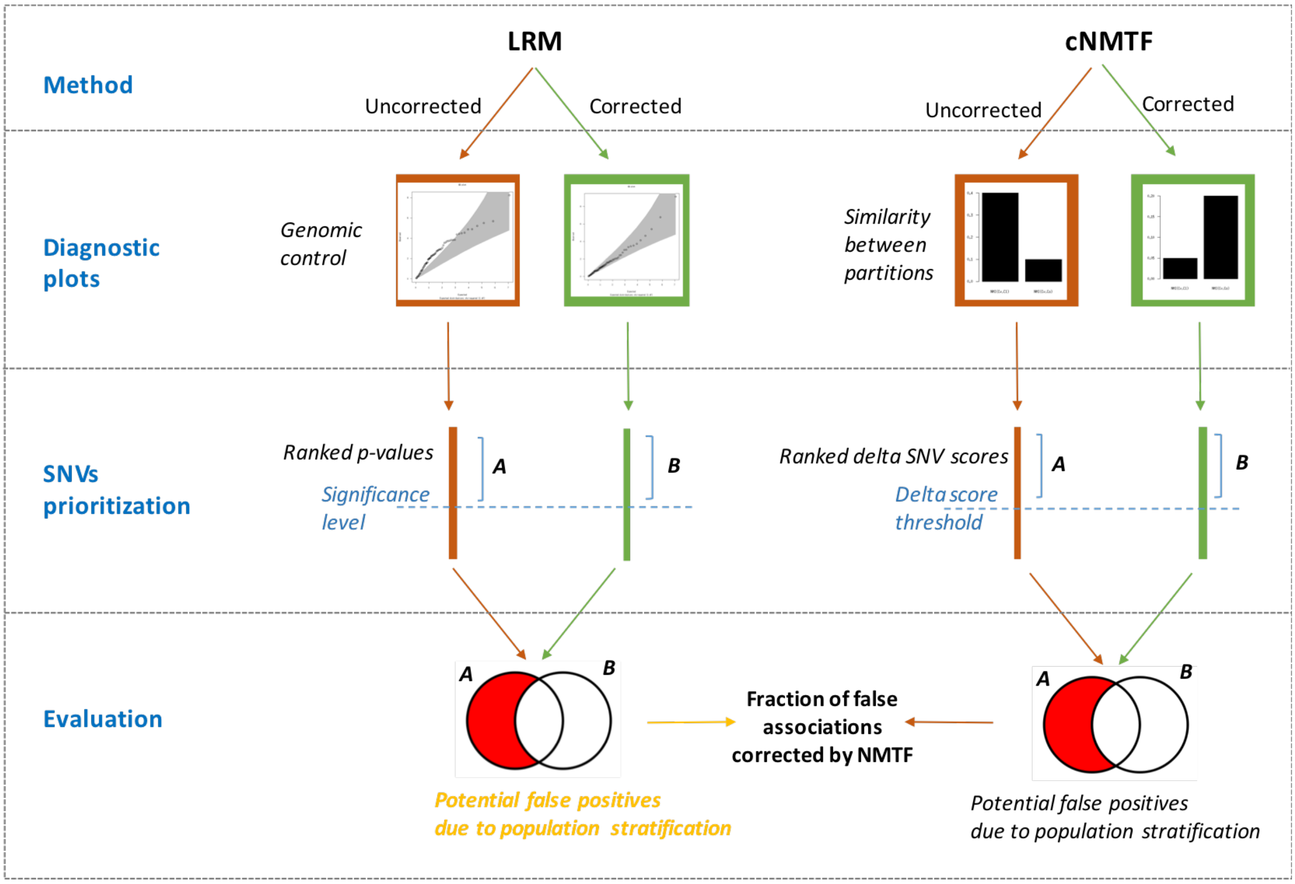

Figure S19: Correcting population structures in LRM and cNMTF. Corrected and uncorrected version of each method are applied in the same data. In LRM, QQ-plots of the p-value distribution are used to diagnose departures from expected quantiles and the genomic control factor is assessed. SNVs are ranked and prioritised if they are above the significance level. In cNMTF, the similarity NMI between partitions of subjects (cluster-population, cluster-phenotype) are used to diagnose confounding effects. SNVs are prioritised if they are beyond the cut-off points of significance. The sets of prioritised variants are compared between corrected and uncorrected versions to define a subset of potential false positive associations (red area of set A).

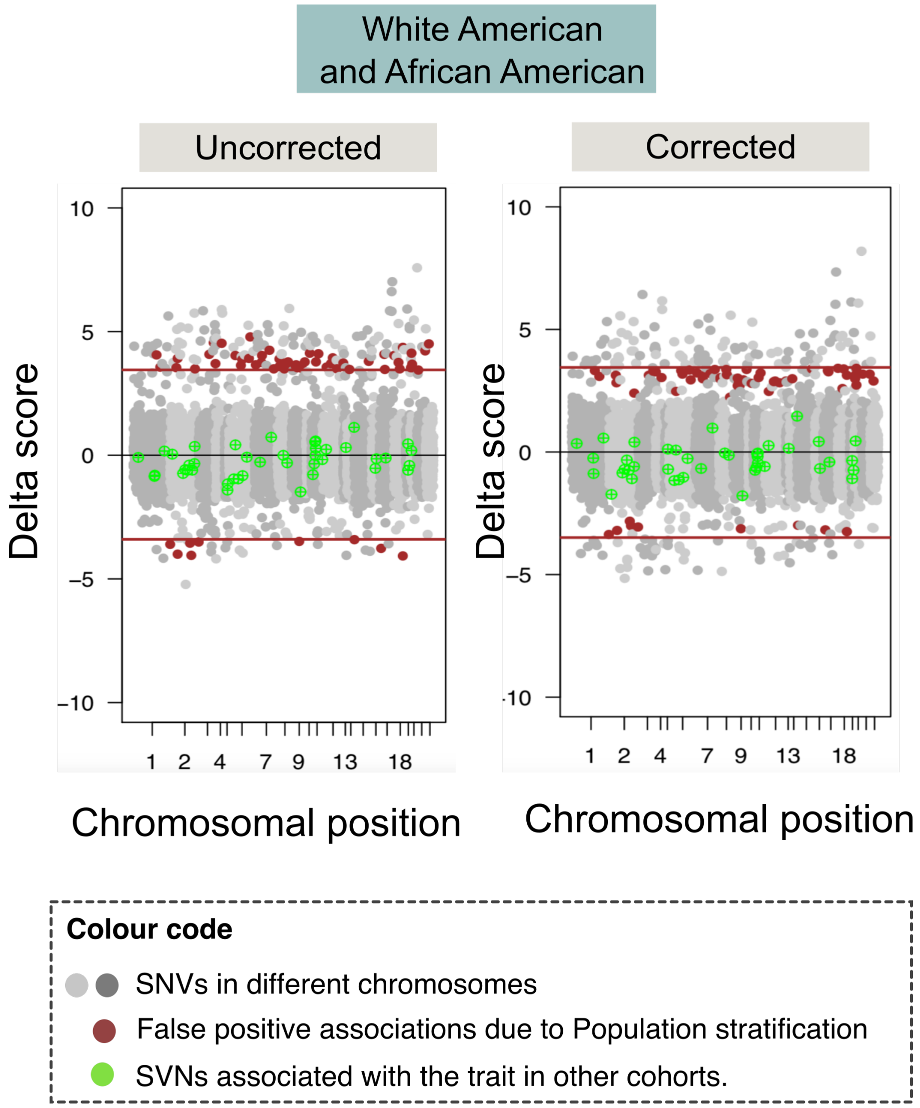

Figure S20: Confounding effect of population structures. We compared the prioritisation results in a mixed cohort of white and African Americans. Variants losing significance between the uncorrected and the corrected results are coloured in red. These variants are potential false positives detected by cNMTF. A total of 62 variants lost significance going from the uncorrected to the corrected versions of cNMTF (red dots); 49 of these variants were also found as false positives between uncorrected and corrected LRMs.

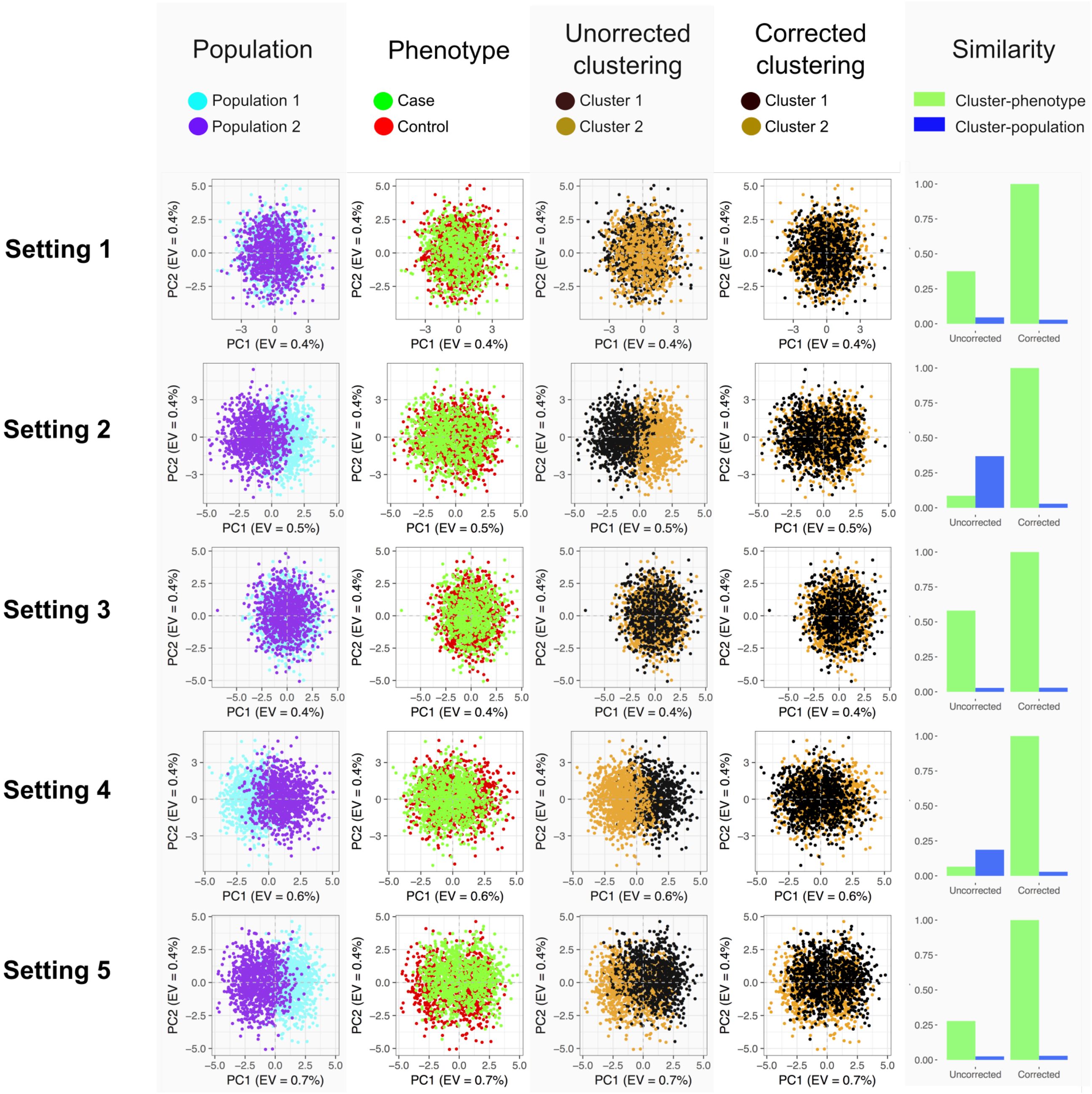

Figure S21: PCA of synthetic GWAS data with population structures. Five synthetic datasets were simulated under different combinations of SNVs as defined in Table S6. The dispersion of subjects in the first two components is depicted for each setting. Subjects are coloured by population labels, phenotype (*i.e*, case or control) and cluster membership using the uncorrected and corrected versions of cNMTF. The last column presents the similarity NMI between partitions of individuals.
